# Supplementary material for: Association of polygenic risk for major psychiatric illness with subcortical volumes and white matter integrity in UK Biobank
Source: Sci Rep. 2017 Feb 10;7:42140. doi: 10.1038/srep42140 (PMC5301496; doi:10.1038/srep42140)
Supplement: Supplementary Method and Tables [file srep42140-s1.pdf]

Association of polygenic risk for major psychiatric illness with subcortical volumes and white matter integrity in UK Biobank

[1]Reus LM, [1]Shen X, [1]Gibson J, [1]Wigmore E, [2]Ligthart L, [1]Adams MJ, [3]Davies G, [3,4]Cox SR, [1,3,4]Hagenaars SP, [3]Bastin ME, [3,4]Deary IJ, [1]Whalley HC*, [1,3]McIntosh AM

[1] Division of Psychiatry, University of Edinburgh, Royal Edinburgh Hospital, Edinburgh, EH10 5HF, United Kingdom;

[2] Department of Biological Psychology, Vrije Universiteit, Amsterdam, the Netherlands;

[3] Centre for Cognitive Ageing and Cognitive Epidemiology, University of Edinburgh, Edinburgh, EH8 9JZ, United Kingdom;

[4] Department of Psychology, University of Edinburgh, Edinburgh, EH8 9JZ, United Kingdom.

* Correspondence to Dr. Heather C. Whalley, heather.whalley@ed.ac.uk.

Supplementary materials

Methods - Image acquisition

Image acquisition have been performed by UK Biobank, and are available on the UK Biobank website (http://www.ukbiobank.ac.uk/), and have been documented previously[1].

T1 and diffusion-weighted (DW) MRI data were acquired in Cheadle Manchester on a Siemens Skyra 3.0T scanner (Siemens Medical Solutions, Erlangen, Germany) with a 32-channel head coil (https://www.healthcare.siemens.com/magnetic-resonance-imaging). The following parameters were used for the T1-weighted sequence: field of view (FOV) = 208x256x256 mm; acquired voxel size = 1x1x1mm; repetition time (TR)/echo time (TE) = 2,000.00/2.01 ms; flip angle= 8º; plane acceleration iPAT = 2; acquisition time = 5.00 minutes. For the DW MRI data, the following parameters were used: FOV = 104x104x72mm; acquired voxel size = 2x2x2mm; TR/TE = 3,600/92ms; flip angle = 78°; acquisition time = 7.00 minutes. 72 slides, aligned to the anterior commissure to the posterior commissure and reversed, were collected along 50 gradient directions with b=1,000 s/mm², b=2,000 s/mm², and ten minimally diffusion (b_0=0 s/mm²) weighted scans.

Methods - Neuroimaging data pre-processing and analysis

T1-weighted and DW volumes were pre-processed and analysed by UK Biobank[1] using Functional Magnetic Resonance Imaging of the Brain (FMRIB) Software Library (FSL, http://fsl.fmrib.ox.ac.uk/fsl)[2]. Pre-processing pipelines are documented on the UK Biobank website (UK Biobank, Brain Imaging Documentation V1.1, http://www.ukbiobank.ac.uk). Manual and automated quality control on the image data was performed prior to data release.

To ensure the anonymity of subjects, the T1-weighted volumes were 'defaced' (*i.e.,* voxels in facial regions were set to zero). Thereafter, gradient distortion correction (GDC) was performed using a tool developed by teams of the Freesurfer and Human Connectome

Project (HCP) (available at https://github.com/Washington-University/Pipelines). Additionally, non-brain tissue was removed using BET (Brain Extraction Tool)[3] and FLIRT (FMRIB's Linear Image Registration Tool)[4,5]. Scans were nonlinearly aligned to Montreal Neurological Institute (MNI) 152 space, and segmented to identify white matter (WM), grey matter (GM) and cerebrospinal fluid (CSF) using FNIRT (FMRIB's Nonlinear Image Registration Tool)[6,7]. Subsequently, total WM, GM, and CSF volume was calculated. Finally, FIRST (FMRIB's Integrated Registration and Segmentation Tool) was used to extract subcortical structures from the GM images[8]. Subcortical volumes were calculated for the following structures: caudate, hippocampus, pallidum, thalamus, amygdala, nucleus accumbens, and putamen bilaterally.

DW volumes were converted from DICOM into 4D Neuroimaging Informatics Technology Initiative (NIfTI) format, and b-vector and b-value files were created using Chris Rorden's dcm2niix conversion tool (https://www.nitrc.org/plugins/mwiki/index.php/dcm2nii:MainPage).

DW volumes were corrected for eddy current distortions (using the Eddy tool)[6,7] and head motion, and outlier-slices were removed. Thereafter, GDC was performed using a similar method as used for the T1-weighted volumes. Mean diffusivity (MD) and FA maps were created by fitting the b= 1,000 s/mm² shell into DTIFIT (DTI fitting tool). FA maps were warped to standard space, followed by a probabilistic tractography analysis. BEDPOSTx (Bayesian Estimation of Diffusion Parameters Obtained using Sampling Techniques) was used to model crossing fibers within each voxel[9]. Consequently, 27 WM tracts (12 bilateral and 3 unilateral WM tracts) were identified by starting at 27 different seed locations (defined by AutoPtx[10]), and fitting voxels that consecutively follow each other's direction using PROBTRACKx.

Tract-averaged water diffusion measures (FA and MD) were calculated for the

following WM tracts: acoustic radiation, cingulum (cingulate gyrus and parahippocampal part), corticospinal tract, forceps major (unilateral), forceps minor (unilateral), inferior fronto-occipital fasciculus, inferior longitudinal fasciculus, medial lemniscus, middle cerebral peduncle (unilateral), thalamic radiation (anterior, superior and posterior), and uncinate fasciculus. MD measures were calculated using the warping and tract defining parameters generated by FA maps. MD and FA are widely-used diffusion measures[11], which describe the directional coherence[12] and mean magnitude of water molecule diffusion independent from direction and tissue type[13] respectively[14].

Results - PGRS and total brain and subcortical volumes

Table S1. Association of PGRS (MDD, SCZ or BP) at other p thresholds with total GM, WM, and CSF, in sample including outliers.

	Including outliers (N = 978)				
	Beta: z ratio (S.D.)	t statistic	p -uncorr.	p-FDR	R^2
**MDD-PGRS**					
GM volume p ≤ 0.01	0.026 (0.027)	0.986	0.324	0.973	0.069
GM volume p ≤ 0.05	0.011 (0.027)	0.410	0.682	1.000	0.012
GM volume p ≤ 0.10	0.006 (0.027)	0.222	0.825	1.000	0.004
GM volume p ≤ 1.00	-0.000 (0.027)	-0.007	0.995	1.000	0.000
WM volume p ≤ 0.01	0.038 (0.026)	1.463	0.144	0.431	0.146
WM volume p ≤ 0.05	0.027 (0.026)	1.024	0.306	0.918	0.073
WM volume p ≤ 0.10	0.004 (0.027)	0.153	0.879	1.000	0.002
WM volume p ≤ 1.00	-0.007 (0.027)	-0.246	0.806	1.000	0.004
CSF volume p ≤ 0.01	0.010 (0.029)	0.360	0.719	1.000	0.011
CSF volume p ≤ 0.05	0.008 (0.030)	0.264	0.792	1.000	0.006
CSF volume p ≤ 0.10	0.005 (0.030)	0.168	0.867	1.000	0.002
CSF volume p ≤ 1.00	0.043 (0.030)	1.424	0.155	0.465	0.182
**SCZ-PGRS**					
GM volume p ≤ 0.01	0.008 (0.027)	0.291	0.771	1.000	0.006
GM volume p ≤ 0.05	-0.021 (0.027)	-0.786	0.432	1.000	0.045
GM volume p ≤ 0.10	-0.018 (0.027)	-0.661	0.509	1.000	0.032
GM volume p ≤ 1.00	-0.013 (0.028)	-0.475	0.635	1.000	0.017
WM volume p ≤ 0.01	-0.007 (0.026)	-0.279	0.781	1.000	0.005
WM volume p ≤ 0.05	-0.019 (0.026)	-0.727	0.467	1.000	0.037
WM volume p ≤ 0.10	-0.009 (0.026)	-0.322	0.747	1.000	0.007
WM volume p ≤ 1.00	-0.002 (0.027)	-0.080	0.936	1.000	0.000
CSF volume p ≤ 0.01	0.046 (0.029)	1.600	0.110	0.330	0.215
CSF volume p ≤ 0.05	0.044 (0.029)	1.490	0.137	0.410	0.193
CSF volume p ≤ 0.10	0.050 (0.030)	1.689	0.092	0.275	0.248
CSF volume p ≤ 1.00	0.046 (0.030)	1.517	0.130	0.389	0.208
**BP-PGRS**					
GM volume p ≤ 0.01	-0.029 (0.027)	-1.069	0.285	0.856	0.081
GM volume p ≤ 0.05	0.002 (0.027)	0.057	0.954	1.000	0.000
GM volume p ≤ 0.10	0.006 (0.027)	0.214	0.830	1.000	0.003
GM volume p ≤ 1.00	-0.001 (0.027)	-0.031	0.975	1.000	0.000
WM volume p ≤ 0.01	-0.023 (0.026)	-0.866	0.387	1.000	0.051
WM volume p ≤ 0.05	0.005 (0.026)	0.189	0.850	1.000	0.002
WM volume p ≤ 0.10	0.005 (0.026)	0.200	0.841	1.000	0.003
WM volume p ≤ 1.00	-0.000 (0.026)	-0.013	0.990	1.000	0.000
CSF volume p ≤ 0.01	0.019 (0.029)	0.658	0.511	1.000	0.037
CSF volume p ≤ 0.05	0.018 (0.029)	0.626	0.532	1.000	0.033
CSF volume p ≤ 0.10	0.004 (0.029)	0.137	0.891	1.000	0.002
CSF volume p ≤ 1.00	-0.007 (0.029)	-0.231	0.817	1.000	0.005

MDD: major depressive disorder, SCZ: schizophrenia, BP: bipolar disorder, GM: grey matter, WM: white matter, CSF:

cerebrospinal fluid, PGRS: polygenic risk scores, uncorr.: uncorrected, FDR: false discovery rate, S.D.: standard deviation. Controlled for age, age^2, gender, genotype batch and array, 15 MDS components and intracranial volume. R^2 = estimate of variance explained by PGRS in %.

Table S2. Association of PGRS for MDD at other p thresholds with subcortical volumes, in sample including outliers.

	Including outliers (N = 978)				
	**Beta: z ratio (S.D.)**	**t statistic**	**p -uncorr.**	**p-FDR**	**R^2**
**MDD-PGRS**					
**Caudate p ≤ 0.01**	0.004 (0.026)	0.141	0.888	1.000	0.001
**Caudate p ≤ 0.05**	0.026 (0.026)	0.970	0.332	1.000	0.066
**Caudate p ≤ 0.10**	0.016 (0.026)	0.633	0.527	1.000	0.027
**Caudate p ≤ 1.00**	0.028 (0.026)	1.048	0.295	1.000	0.077
**Hippocampus p ≤ 0.01**	0.005 (0.025)	0.212	0.832	1.000	0.003
**Hippocampus p ≤ 0.05**	-0.002 (0.026)	-0.082	0.935	1.000	0.000
**Hippocampus p ≤ 0.10**	0.010 (0.025)	0.415	0.678	1.000	0.011
**Hippocampus p ≤ 1.00**	0.000 (0.026)	0.007	0.994	1.000	0.000
**Pallidum p ≤ 0.01**	-0.011 (0.026)	-0.422	0.673	1.000	0.012
**Pallidum p ≤ 0.05**	0.003 (0.027)	0.107	0.915	1.000	0.001
**Pallidum p ≤ 0.10**	0.009 (0.026)	0.326	0.744	1.000	0.007
**Pallidum p ≤ 1.00**	-0.006 (0.027)	-0.218	0.827	1.000	0.003
**Thalamus p ≤ 0.01**	0.003 (0.020)	0.169	0.866	1.000	0.001
**Thalamus p ≤ 0.05**	-0.012 (0.020)	-0.611	0.541	1.000	0.015
**Thalamus p ≤ 0.10**	-0.004 (0.020)	-0.196	0.844	1.000	0.001
**Thalamus p ≤ 1.00**	-0.013 (0.020)	-0.640	0.522	1.000	0.017
**Amygdala p ≤ 0.01**	-0.020 (0.026)	-0.763	0.445	1.000	0.039
**Amygdala p ≤ 0.05**	-0.000 (0.027)	-0.010	0.992	1.000	0.000
**Amygdala p ≤ 0.10**	-0.009 (0.026)	-0.359	0.720	1.000	0.009
**Amygdala p ≤ 1.00**	0.010 (0.027)	0.385	0.700	1.000	0.010
**Nucleus accumbens p ≤ 0.01**	0.000 (0.025)	0.005	0.996	1.000	0.000
**Nucleus accumbens p ≤ 0.05**	-0.019 (0.026)	-0.742	0.458	1.000	0.038
**Nucleus accumbens p ≤ 0.10**	0.014 (0.026)	0.526	0.599	1.000	0.018
**Nucleus accumbens p ≤ 1.00**	-0.019 (0.026)	-0.717	0.474	1.000	0.035
**Putamen p ≤ 0.01**	-0.008 (0.022)	-0.391	0.696	1.000	0.007
**Putamen p ≤ 0.05**	0.007 (0.022)	0.309	0.757	1.000	0.005
**Putamen p ≤ 0.10**	0.006 (0.022)	0.278	0.781	1.000	0.004
**Putamen p ≤ 1.00**	0.007 (0.022)	0.296	0.767	1.000	0.004

MDD: major depressive disorder, PGRS: polygenic risk scores, uncorr.: uncorrected, FDR: false discovery rate, S.D.: standard deviation. Controlled for age, age^2, gender, genotype batch and array, 15 MDS components, intracranial volume and side of hemisphere. R^2 = estimate of variance explained by PGRS in %.

Table S3. Association of PGRS for SCZ at other p thresholds with subcortical volumes, in sample including outliers.

	Including outliers (N = 978)				
	**Beta: z ratio (S.D.)**	**t statistic**	**p -uncorr.**	**p-FDR**	R^2
**SCZ-PGRS**					
**Caudate p ≤ 0.01**	0.006 (0.026)	0.246	0.806	1.000	0.004
**Caudate p ≤ 0.05**	-0.003 (0.026)	-0.101	0.919	1.000	0.001
**Caudate p ≤ 0.10**	0.009 (0.026)	0.350	0.726	1.000	0.008
**Caudate p ≤ 1.00**	-0.006 (0.026)	-0.238	0.812	1.000	0.004
**Hippocampus p ≤ 0.01**	-0.014 (0.025)	-0.566	0.572	1.000	0.020
**Hippocampus p ≤ 0.05**	-0.001 (0.025)	-0.044	0.965	1.000	0.000
**Hippocampus p ≤ 0.10**	-0.001 (0.025)	-0.030	0.976	1.000	0.000
**Hippocampus p ≤ 1.00**	0.006 (0.025)	0.233	0.816	1.000	0.004
**Pallidum p ≤ 0.01**	-0.017 (0.026)	-0.650	0.516	1.000	0.028
**Pallidum p ≤ 0.05**	-0.015 (0.027)	-0.578	0.564	1.000	0.024
**Pallidum p ≤ 0.10**	-0.019 (0.026)	-0.719	0.472	1.000	0.036
**Pallidum p ≤ 1.00**	-0.017 (0.027)	-0.633	0.527	1.000	0.028
**Thalamus p ≤ 0.01**	-0.029 (0.020)	-1.477	0.140	0.979	0.083
**Thalamus p ≤ 0.05**	-0.039 (0.020)	-1.929	0.052	0.378	0.149
**Thalamus p ≤ 0.10**	-0.043 (0.020)	-2.170	0.030	0.212	0.183
**Thalamus p ≤ 1.00**	-0.040 (0.020)	-1.979	0.048	0.337	0.157
**Amygdala p ≤ 0.01**	0.013 (0.026)	0.517	0.606	1.000	0.018
**Amygdala p ≤ 0.05**	0.015 (0.026)	0.558	0.577	1.000	0.022
**Amygdala p ≤ 0.10**	0.023 (0.026)	0.896	0.370	1.000	0.055
**Amygdala p ≤ 1.00**	0.017 (0.026)	0.629	0.530	1.000	0.028
**Nucleus accumbens p ≤ 0.01**	-0.011 (0.026)	-0.449	0.653	1.000	0.013
**Nucleus accumbens p ≤ 0.05**	-0.024 (0.026)	-0.924	0.356	1.000	0.058
**Nucleus accumbens p ≤ 0.10**	-0.036 (0.026)	-1.412	0.158	1.000	0.132
**Nucleus accumbens p ≤ 1.00**	-0.023 (0.026)	-0.882	0.378	1.000	0.053
**Putamen p ≤ 0.01**	-0.006 (0.022)	-0.263	0.793	1.000	0.003
**Putamen p ≤ 0.05**	0.005 (0.022)	0.211	0.833	1.000	0.002
**Putamen p ≤ 0.10**	-0.001 (0.022)	-0.030	0.976	1.000	0.000
**Putamen p ≤ 1.00**	0.000 (0.022)	0.015	0.988	1.000	0.000

SCZ: schizophrenia, PGRS: polygenic risk scores, uncorr.: uncorrected, FDR: false discovery rate, S.D.: standard deviation. Controlled for age, age^2, gender, genotype batch and array, 15 MDS components, intracranial volume and side of hemisphere.

R^2 = estimate of variance explained by PGRS in %.

Table S4. Association of PGRS for BP at other p thresholds with subcortical volumes, in sample including outliers.

	Including outliers (N = 978)				
	**Beta: z ratio (S.D.)**	**t statistic**	**p -uncorr.**	**p-FDR**	**R^2**
**BP-PGRS**					
**Caudate p ≤ 0.01**	0.034 (0.026)	1.295	0.196	1.000	0.113
**Caudate p ≤ 0.05**	0.017 (0.026)	0.653	0.514	1.000	0.029
**Caudate p ≤ 0.10**	0.019 (0.026)	0.747	0.455	1.000	0.037
**Caudate p ≤ 1.00**	0.022 (0.026)	0.852	0.394	1.000	0.049
**Hippocampus p ≤ 0.01**	0.006 (0.025)	0.235	0.814	1.000	0.003
**Hippocampus p ≤ 0.05**	0.024 (0.025)	0.963	0.336	1.000	0.058
**Hippocampus p ≤ 0.10**	0.016 (0.025)	0.658	0.511	1.000	0.027
**Hippocampus p ≤ 1.00**	0.030 (0.025)	1.177	0.239	1.000	0.087
**Pallidum p ≤ 0.01**	0.038 (0.026)	1.449	0.148	1.000	0.143
**Pallidum p ≤ 0.05**	-0.000 (0.026)	-0.009	0.993	1.000	0.000
**Pallidum p ≤ 0.10**	0.023 (0.026)	0.871	0.384	1.000	0.051
**Pallidum p ≤ 1.00**	0.005 (0.026)	0.179	0.858	1.000	0.002
**Thalamus p ≤ 0.01**	-0.005 (0.020)	-0.251	0.802	1.000	0.002
**Thalamus p ≤ 0.05**	0.010 (0.020)	0.517	0.605	1.000	0.010
**Thalamus p ≤ 0.10**	-0.009 (0.020)	-0.447	0.655	1.000	0.008
**Thalamus p ≤ 1.00**	0.009 (0.020)	0.432	0.666	1.000	0.007
**Amygdala p ≤ 0.01**	-0.006 (0.026)	-0.225	0.822	1.000	0.003
**Amygdala p ≤ 0.05**	0.030 (0.026)	1.158	0.247	1.000	0.091
**Amygdala p ≤ 0.10**	-0.001 (0.026)	-0.020	0.984	1.000	0.000
**Amygdala p ≤ 1.00**	0.029 (0.026)	1.130	0.259	1.000	0.087
**Nucleus accumbens p ≤ 0.01**	0.025 (0.026)	0.987	0.324	1.000	0.064
**Nucleus accumbens p ≤ 0.05**	-0.008 (0.026)	-0.296	0.767	1.000	0.006
**Nucleus accumbens p ≤ 0.10**	0.000 (0.026)	0.013	0.989	1.000	0.000
**Nucleus accumbens p ≤ 1.00**	-0.007 (0.026)	-0.262	0.793	1.000	0.005
**Putamen p ≤ 0.01**	0.001 (0.022)	0.060	0.953	1.000	0.000
**Putamen p ≤ 0.05**	0.025 (0.022)	1.133	0.257	1.000	0.061
**Putamen p ≤ 0.10**	0.027 (0.022)	1.229	0.219	1.000	0.071
**Putamen p ≤ 1.00**	0.024 (0.022)	1.114	0.265	1.000	0.059

BP: bipolar disorder, PGRS: polygenic risk scores, uncorr.: uncorrected, FDR: false discovery rate, S.D.: standard deviation. Controlled for age, age^2, gender, genotype batch and array, 15 MDS components, intracranial volume and side of hemisphere.

R^2 = estimate of variance explained by PGRS in %.

Results - PGRS and diffusion measures

Table S5. Association of PGRS (MDD, SCZ or BP) at other p thresholds with *g*FA and *g*MD, in sample including outliers.

	Including outliers (N = 816)					Including outliers (N = 816)			
	**Beta: z ratio (S.D.)**	**t statistic**	**p**	**R^2**		**Beta: z ratio (S.D.)**	**t statistic**	**p**	**R^2**
**MDD-PGRS**					**MDD-PGRS**				
***g*FA p ≤ 0.01**	0.030 (0.035)	0.843	0.399	0.088	***g*MD p ≤ 0.01**	-0.056 (0.034)	-1.649	0.099 #	0.316
***g*FA p ≤ 0.05**	0.041 (0.036)	1.153	0.249	0.170	***g*MD p ≤ 0.05**	-0.065 (0.035)	-1.895	0.059 #	0.429
***g*FA p ≤ 0.10**	0.013 (0.036)	0.370	0.711	0.018	***g*MD p ≤ 0.10**	-0.052 (0.035)	-1.491	0.136	0.268
***g*FA p ≤ 1.00**	0.017 (0.036)	0.465	0.642	0.029	***g*MD p ≤ 1.00**	-0.044 (0.035)	-1.239	0.216	0.192
**SCZ-PGRS**					**SCZ-PGRS**				
***g*FA p ≤ 0.01**	0.020 (0.035)	0.561	0.575	0.039	***g*MD p ≤ 0.01**	-0.035 (0.034)	-1.021	0.307	0.121
***g*FA p ≤ 0.05**	-0.004 (0.036)	-0.121	0.903	0.002	***g*MD p ≤ 0.05**	-0.014 (0.035)	-0.403	0.687	0.019
***g*FA p ≤ 0.10**	-0.030 (0.036)	-0.845	0.398	0.091	***g*MD p ≤ 0.10**	-0.006 (0.035)	-0.185	0.854	0.004
***g*FA p ≤ 1.00**	-0.030 (0.036)	-0.831	0.406	0.091	***g*MD p ≤ 1.00**	-0.012 (0.035)	-0.338	0.736	0.014
**BP-PGRS**					**BP-PGRS**				
***g*FA p ≤ 0.01**	0.035 (0.035)	1.005	0.315	0.124	***g*MD p ≤ 0.01**	-0.040 (0.034)	-1.189	0.235	0.163
***g*FA p ≤ 0.05**	0.001 (0.035)	0.020	0.984	0.000	***g*MD p ≤ 0.05**	-0.013 (0.034)	-0.387	0.699	0.017
***g*FA p ≤ 0.10**	0.014 (0.035)	0.404	0.686	0.020	***g*MD p ≤ 0.10**	-0.008 (0.034)	-0.226	0.821	0.006
***g*FA p ≤ 1.00**	-0.004 (0.036)	-0.114	0.909	0.002	***g*MD p ≤ 1.00**	0.009 (0.035)	0.255	0.799	0.008

FA: fractional anisotropy, MD: mean diffusivity, g: general factor, MDD: major depressive disorder, SCZ: schizophrenia, BP: bipolar disorder, PGRS: polygenic risk scores, S.D.: standard deviation. Controlled for age, age^2 and gender, genotype batch and array, and 15 MDS components. R^2 = estimate of variance explained by PGRS in %. # depicts trendwise associations ($p < 0.10$).

Table S6. Association of PGRS (MDD, SCZ or BP) at other p thresholds with *g*FA and *g*MD for association, projection and thalamic WM fibers, in sample including outliers.

	Including outliers (N = 816)					Including outliers (N = 816)			
	**Beta: z ratio (S.D.)**	**t statistic**	**p**	**R^2**		**Beta: z ratio (S.D.)**	**t statistic**	**p**	**R^2**
**MDD-PGRS**					**MDD-PGRS**				
***g*FA Association p ≤ 0.01**	0.031 (0.035)	0.876	0.381	0.095	***g*MD Association p ≤ 0.01**	-0.054 (0.034)	-1.571	0.116	0.293
***g*FA Association p ≤ 0.05**	0.046 (0.036)	1.288	0.198	0.211	***g*MD Association p ≤ 0.05**	-0.065 (0.035)	-1.868	0.062 #	0.426
***g*FA Association p ≤ 0.10**	0.019 (0.036)	0.544	0.587	0.038	***g*MD Association p ≤ 0.10**	-0.049 (0.035)	-1.396	0.163	0.241
***g*FA Association p ≤ 1.00**	0.039 (0.036)	1.068	0.286	0.151	***g*MD Association p ≤ 1.00**	-0.049 (0.036)	-1.363	0.173	0.237
***g*FA Projection p ≤ 0.01**	0.034 (0.035)	0.989	0.323	0.118	***g*MD Projection p ≤ 0.01**	0.030 (0.035)	0.848	0.397	0.087
***g*FA Projection p ≤ 0.05**	0.040 (0.035)	1.120	0.263	0.156	***g*MD Projection p ≤ 0.05**	0.024 (0.035)	0.665	0.506	0.055
***g*FA Projection p ≤ 0.10**	0.031 (0.035)	0.882	0.378	0.098	***g*MD Projection p ≤ 0.10**	0.012 (0.036)	0.334	0.738	0.014
***g*FA Projection p ≤ 1.00**	-0.015 (0.036)	-0.405	0.685	0.021	***g*MD Projection p ≤ 1.00**	-0.024 (0.036)	-0.662	0.508	0.057
***g*FA Thalamic p ≤ 0.01**	0.020 (0.036)	0.569	0.570	0.041	***g*MD Thalamic p ≤ 0.01**	-0.055 (0.033)	-1.684	0.093 #	0.300
***g*FA Thalamic p ≤ 0.05**	0.037 (0.036)	1.035	0.301	0.140	***g*MD Thalamic p ≤ 0.05**	-0.063 (0.033)	-1.912	0.056 #	0.399
***g*FA Thalamic p ≤ 0.10**	0.001 (0.036)	0.021	0.983	0.000	***g*MD Thalamic p ≤ 0.10**	-0.044 (0.033)	-1.332	0.183	0.196
***g*FA Thalamic p ≤ 1.00**	0.004 (0.037)	0.096	0.924	0.001	***g*MD Thalamic p ≤ 1.00**	-0.031 (0.034)	-0.924	0.356	0.097
**SCZ-PGRS**					**SCZ-PGRS**				
***g*FA Association p ≤ 0.01**	0.027 (0.035)	0.769	0.442	0.073	***g*MD Association p ≤ 0.01**	-0.015 (0.034)	-0.435	0.664	0.022
***g*FA Association p ≤ 0.05**	-0.004 (0.036)	-0.112	0.911	0.002	***g*MD Association p ≤ 0.05**	0.004 (0.035)	0.113	0.910	0.002
***g*FA Association p ≤ 0.10**	-0.024 (0.036)	-0.683	0.495	0.059	***g*MD Association p ≤ 0.10**	0.011 (0.035)	0.308	0.758	0.012
***g*FA Association p ≤ 1.00**	-0.023 (0.036)	-0.636	0.525	0.053	***g*MD Association p ≤ 1.00**	-0.001 (0.036)	-0.026	0.979	0.000
***g*FA Projection p ≤ 0.01**	0.006 (0.035)	0.181	0.857	0.004	***g*MD Projection p ≤ 0.01**	0.010 (0.035)	0.302	0.763	0.011
***g*FA Projection p ≤ 0.05**	0.001 (0.035)	0.016	0.987	0.000	***g*MD Projection p ≤ 0.05**	0.002 (0.035)	0.064	0.949	0.001
***g*FA Projection p ≤ 0.10**	-0.025 (0.035)	-0.707	0.480	0.062	***g*MD Projection p ≤ 0.10**	-0.029 (0.035)	-0.827	0.409	0.085
***g*FA Projection p ≤ 1.00**	-0.028 (0.036)	-0.785	0.433	0.079	***g*MD Projection p ≤ 1.00**	-0.033 (0.036)	-0.915	0.360	0.108
***g*FA Thalamic p ≤ 0.01**	0.014 (0.036)	0.408	0.683	0.021	***g*MD Thalamic p ≤ 0.01**	-0.056 (0.032)	-1.731	0.084 #	0.315
***g*FA Thalamic p ≤ 0.05**	-0.005 (0.036)	-0.146	0.884	0.003	***g*MD Thalamic p ≤ 0.05**	-0.032 (0.033)	-0.959	0.338	0.100
***g*FA Thalamic p ≤ 0.10**	-0.031 (0.036)	-0.870	0.385	0.098	***g*MD Thalamic p ≤ 0.10**	-0.025 (0.033)	-0.751	0.453	0.061
***g*FA Thalamic p ≤ 1.00**	-0.034 (0.037)	-0.923	0.356	0.115	***g*MD Thalamic p ≤ 1.00**	-0.019 (0.034)	-0.578	0.563	0.038
**BP-PGRS**					**BP-PGRS**				

***g*FA Association p ≤ 0.01**	0.032 (0.035)	0.926	0.355	0.105	***g*MD Association p ≤ 0.01**	-0.028 (0.034)	-0.810	0.418	0.078
***g*FA Association p ≤ 0.05**	0.003 (0.035)	0.087	0.930	0.001	***g*MD Association p ≤ 0.05**	-0.011 (0.035)	-0.327	0.744	0.013
***g*FA Association p ≤ 0.10**	0.018 (0.035)	0.508	0.612	0.032	***g*MD Association p ≤ 0.10**	-0.006 (0.035)	-0.172	0.864	0.004
***g*FA Association p ≤ 1.00**	-0.007 (0.036)	-0.210	0.834	0.006	***g*MD Association p ≤ 1.00**	0.013 (0.035)	0.365	0.715	0.016
***g*FA Projection p ≤ 0.01**	0.019 (0.035)	0.548	0.584	0.036	***g*MD Projection p ≤ 0.01**	0.016 (0.035)	0.469	0.639	0.027
***g*FA Projection p ≤ 0.05**	-0.016 (0.035)	-0.454	0.650	0.025	***g*MD Projection p ≤ 0.05**	-0.014 (0.035)	-0.411	0.681	0.021
***g*FA Projection p ≤ 0.10**	-0.002 (0.035)	-0.045	0.964	0.000	***g*MD Projection p ≤ 0.10**	-0.000 (0.035)	-0.007	0.995	0.000
***g*FA Projection p ≤ 1.00**	-0.021 (0.035)	-0.591	0.555	0.043	***g*MD Projection p ≤ 1.00**	-0.018 (0.035)	-0.503	0.615	0.031
***g*FA Thalamic p ≤ 0.01**	0.049 (0.035)	1.383	0.167	0.240	***g*MD Thalamic p ≤ 0.01**	-0.041 (0.032)	-1.251	0.211	0.165
***g*FA Thalamic p ≤ 0.05**	0.005 (0.036)	0.138	0.890	0.002	***g*MD Thalamic p ≤ 0.05**	-0.014 (0.033)	-0.445	0.657	0.021
***g*FA Thalamic p ≤ 0.10**	0.012 (0.036)	0.343	0.732	0.015	***g*MD Thalamic p ≤ 0.10**	-0.004 (0.033)	-0.120	0.904	0.002
***g*FA Thalamic p ≤ 1.00**	0.012 (0.036)	0.327	0.744	0.014	***g*MD Thalamic p ≤ 1.00**	0.005 (0.033)	0.138	0.891	0.002

FA: fractional anisotropy, WM: white matter, g: general factor, MDD: major depressive disorder, SCZ: schizophrenia, BP: bipolar disorder, PGRS: polygenic risk scores, S.D.: standard deviation. Controlled for age, age^2 and gender, genotype batch and array, and 15 MDS components. R^2 = estimate of variance explained by PGRS in %. # depicts trendwise associations ($p < 0.10$).

Table S7. Association of PGRS (MDD, SCZ and BP) at p≤ 0.5 with FA, in sample including and excluding outliers.

	Including outliers (N = 816)					Excluding outliers (N = 733)				
	**Beta: z ratio (S.D.)**	**t statistic**	**p -uncorr.**	**p-FDR**	**R^2**	**Beta: z ratio (S.D.)**	**t statistic**	**p -uncorr**	**p-FDR**	**R^2**
**MDD-PGRS**										
**Acoustic radiation**	0.006 (0.031)	0.187	0.852	1.000	0.003	0.021 (0.033)	0.644	0.520	1.000	0.045
**Cingulum (cingulate gyrus)**	-0.061 (0.031)	-1.975	0.049	0.584	0.376	-0.054 (0.033)	-1.664	0.097	1.000	0.294
**Cingulum (parahippocampal part)**	0.030 (0.031)	0.965	0.335	1.000	0.088	0.024 (0.033)	0.746	0.456	1.000	0.059
**Corticospinal tract**	-0.028 (0.033)	-0.849	0.396	1.000	0.078	-0.011 (0.034)	-0.332	0.740	1.000	0.013
**Forceps major (unilateral)**	-0.009 (0.038)	-0.246	0.805	1.000	0.009	0.011 (0.040)	0.267	0.789	1.000	0.011
**Forceps minor (unilateral)**	-0.028 (0.036)	-0.779	0.436	1.000	0.079	0.008 (0.038)	0.210	0.834	1.000	0.006
**Inferior fronto-occipital fasciculus**	-0.065 (0.034)	-1.903	0.057	0.688	0.417	-0.062 (0.035)	-1.756	0.080	0.955	0.387
**Inferior longitudinal fasciculus**	-0.040 (0.034)	-1.173	0.241	1.000	0.158	-0.029 (0.036)	-0.825	0.410	1.000	0.086
**Medial lemniscus**	0.017 (0.030)	0.568	0.570	1.000	0.029	0.033 (0.031)	1.055	0.292	1.000	0.110
**Middle cerebellar peduncle (unilateral)**	-0.013 (0.037)	-0.352	0.725	1.000	0.017	-0.035 (0.039)	-0.918	0.359	1.000	0.126
**Superior longitudinal fasciculus**	-0.044 (0.034)	-1.287	0.199	1.000	0.189	-0.025 (0.036)	-0.686	0.493	1.000	0.060
**Thalamic radiation (anterior)**	-0.065 (0.033)	-1.933	0.054	0.643	0.416	-0.053 (0.035)	-1.501	0.134	1.000	0.281
**Thalamic radiation (posterior)**	-0.030 (0.033)	-0.890	0.374	1.000	0.088	-0.029 (0.035)	-0.828	0.408	1.000	0.082
**Thalamic radiation (superior)**	0.010 (0.034)	0.282	0.778	1.000	0.009	0.026 (0.036)	0.743	0.458	1.000	0.070
**Uncinate fasciculus**	-0.017 (0.032)	-0.521	0.603	1.000	0.027	-0.005 (0.033)	-0.165	0.869	1.000	0.003
**SCZ-PGRS**										
**Acoustic radiation**	0.026 (0.031)	0.833	0.405	1.000	0.068	0.031 (0.033)	0.926	0.355	1.000	0.094
**Cingulum (cingulate gyrus)**	-0.027 (0.031)	-0.854	0.393	1.000	0.072	-0.028 (0.033)	-0.863	0.388	1.000	0.081
**Cingulum (parahippocampal part)**	-0.076 (0.031)	-2.479	0.013	0.161	0.581	-0.068 (0.033)	-2.067	0.039	0.469	0.460
**Corticospinal tract**	-0.004 (0.033)	-0.121	0.904	1.000	0.002	-0.008 (0.035)	-0.216	0.829	1.000	0.006
**Forceps major (unilateral)**	0.021 (0.038)	0.562	0.574	1.000	0.045	0.021 (0.040)	0.526	0.599	1.000	0.045
**Forceps minor (unilateral)**	0.003 (0.036)	0.093	0.926	1.000	0.001	0.031 (0.038)	0.811	0.418	1.000	0.097
**Inferior fronto-occipital fasciculus**	0.025 (0.034)	0.734	0.463	1.000	0.063	0.038 (0.036)	1.060	0.289	1.000	0.144
**Inferior longitudinal fasciculus**	0.019 (0.034)	0.554	0.580	1.000	0.036	0.034 (0.036)	0.945	0.345	1.000	0.115
**Medial lemniscus**	-0.018 (0.030)	-0.603	0.547	1.000	0.033	-0.016 (0.032)	-0.515	0.607	1.000	0.027
**Middle cerebellar peduncle (unilateral)**	-0.005 (0.037)	-0.136	0.892	1.000	0.002	0.000 (0.039)	0.013	0.990	1.000	0.000
**Superior longitudinal fasciculus**	0.011 (0.034)	0.312	0.755	1.000	0.011	0.019 (0.036)	0.516	0.606	1.000	0.035

**Thalamic radiation (anterior)**	0.021 (0.034)	0.616	0.538	1.000	0.043	0.031 (0.036)	0.870	0.385	1.000	0.096
**Thalamic radiation (posterior)**	-0.018 (0.034)	-0.546	0.585	1.000	0.034	-0.005 (0.035)	-0.154	0.878	1.000	0.003
**Thalamic radiation (superior)**	0.028 (0.034)	0.829	0.407	1.000	0.080	0.043 (0.036)	1.202	0.230	1.000	0.186
**Uncinate fasciculus**	0.029 (0.032)	0.904	0.366	1.000	0.084	0.028 (0.034)	0.838	0.402	1.000	0.079
**BP-PGRS**										
**Acoustic radiation**	-0.004 (0.031)	-0.116	0.908	1.000	0.001	0.006 (0.033)	0.198	0.843	1.000	0.004
**Cingulum (cingulate gyrus)**	-0.033 (0.031)	-1.075	0.283	1.000	0.111	-0.029 (0.033)	-0.884	0.377	1.000	0.083
**Cingulum (parahippocampal part)**	-0.006 (0.031)	-0.208	0.835	1.000	0.004	-0.010 (0.033)	-0.301	0.763	1.000	0.010
**Corticospinal tract**	0.032 (0.033)	0.968	0.334	1.000	0.100	0.029 (0.034)	0.844	0.399	1.000	0.084
**Forceps major (unilateral)**	0.032 (0.037)	0.866	0.387	1.000	0.103	0.032 (0.039)	0.816	0.415	1.000	0.102
**Forceps minor (unilateral)**	-0.005 (0.035)	-0.150	0.881	1.000	0.003	0.001 (0.037)	0.021	0.983	1.000	0.000
**Inferior fronto-occipital fasciculus**	-0.003 (0.034)	-0.097	0.923	1.000	0.001	0.031 (0.035)	0.886	0.376	1.000	0.098
**Inferior longitudinal fasciculus**	0.007 (0.034)	0.218	0.827	1.000	0.005	0.035 (0.035)	0.986	0.324	1.000	0.122
**Medial lemniscus**	0.048 (0.030)	1.632	0.103	1.000	0.233	0.048 (0.031)	1.532	0.126	1.000	0.230
**Middle cerebellar peduncle (unilateral)**	0.023 (0.036)	0.648	0.517	1.000	0.054	-0.006 (0.038)	-0.159	0.874	1.000	0.004
**Superior longitudinal fasciculus**	-0.003 (0.034)	-0.078	0.938	1.000	0.001	0.011 (0.036)	0.316	0.752	1.000	0.013
**Thalamic radiation (anterior)**	0.001 (0.033)	0.044	0.965	1.000	0.000	0.027 (0.035)	0.762	0.446	1.000	0.072
**Thalamic radiation (posterior)**	-0.010 (0.033)	-0.302	0.763	1.000	0.010	0.032 (0.035)	0.913	0.362	1.000	0.100
**Thalamic radiation (superior)**	0.017 (0.034)	0.489	0.625	1.000	0.027	0.022 (0.036)	0.615	0.539	1.000	0.048
**Uncinate fasciculus**	0.022 (0.032)	0.707	0.480	1.000	0.050	0.034 (0.033)	1.030	0.304	1.000	0.116

MDD: major depressive disorder, SCZ: schizophrenia, BP: bipolar disorder, PGRS: polygenic risk scores, uncorr.: uncorrected, FDR: false discovery rate, S.D.: standard deviation.

Controlled for age, age^2, gender, genotype batch and array, 15 MDS components and side of hemisphere.

* Analyses performed separately for association of SCZ-PGRS with FA values of the cingulum (parahippocampal) for the analysis including outliers.

R^2 = estimate of variance explained by PGRS in %.

Table S8. Association of PGRS (MDD, SCZ or BP) at other p thresholds with FA, in sample including outliers.

	Including outliers (N = 816)				
	**Beta: z ratio (S.D.)**	**t statistic**	**p -uncorr.**	**p-FDR**	**R^2**
**MDD-PGRS**					
**Acoustic radiation p ≤ 0.01**	-0.051 (0.031)	-1.659	0.097	1.000	0.262
**Acoustic radiation p ≤ 0.05**	0.004 (0.031)	0.123	0.903	1.000	0.001
**Acoustic radiation p ≤ 0.10**	0.010 (0.031)	0.323	0.747	1.000	0.010
**Acoustic radiation p ≤ 1.00**	0.019 (0.032)	0.605	0.545	1.000	0.037
**Cingulum (cingulate gyrus) p ≤ 0.01**	-0.060 (0.031)	-1.951	0.051	0.771	0.359
**Cingulum (cingulate gyrus) p ≤ 0.05**	-0.062 (0.031)	-1.987	0.047	0.709	0.379
**Cingulum (cingulate gyrus) p ≤ 0.10**	-0.020 (0.031)	-0.650	0.516	1.000	0.041
**Cingulum (cingulate gyrus) p ≤ 1.00**	-0.057 (0.032)	-1.801	0.072	1.000	0.327
**Cingulum (parahippocampal part) p ≤ 0.01**	-0.003 (0.030)	-0.093	0.926	1.000	0.001
**Cingulum (parahippocampal part) p ≤ 0.05**	0.029 (0.030)	0.948	0.343	1.000	0.084
**Cingulum (parahippocampal part) p ≤ 0.10**	0.030 (0.031)	0.972	0.331	1.000	0.089
**Cingulum (parahippocampal part) p ≤ 1.00**	0.010 (0.031)	0.330	0.741	1.000	0.011
**Corticospinal tract p ≤ 0.01**	-0.031 (0.032)	-0.942	0.347	1.000	0.093
**Corticospinal tract p ≤ 0.05**	-0.028 (0.033)	-0.849	0.396	1.000	0.077
**Corticospinal tract p ≤ 0.10**	-0.018 (0.033)	-0.561	0.575	1.000	0.034
**Corticospinal tract p ≤ 1.00**	0.025 (0.034)	0.737	0.461	1.000	0.061
**Forceps major (unilateral) p ≤ 0.01**	-0.022 (0.036)	-0.630	0.529	1.000	0.050
**Forceps major (unilateral) p ≤ 0.05**	-0.060 (0.036)	-1.678	0.094	1.000	0.362
**Forceps major (unilateral) p ≤ 0.10**	-0.048 (0.036)	-1.329	0.184	1.000	0.229
**Forceps major (unilateral) p ≤ 1.00**	-0.017 (0.037)	-0.463	0.643	1.000	0.029
**Forceps minor (unilateral) p ≤ 0.01**	-0.007 (0.034)	-0.191	0.848	1.000	0.004
**Forceps minor (unilateral) p ≤ 0.05**	-0.023 (0.035)	-0.662	0.508	1.000	0.052
**Forceps minor (unilateral) p ≤ 0.10**	-0.001 (0.035)	-0.020	0.984	1.000	0.000
**Forceps minor (unilateral) p ≤ 1.00**	-0.029 (0.035)	-0.815	0.415	1.000	0.083
**Inferior fronto-occipital fasciculus p ≤ 0.01**	-0.036 (0.034)	-1.069	0.285	1.000	0.129
**Inferior fronto-occipital fasciculus p ≤ 0.05**	-0.066 (0.034)	-1.937	0.053	0.797	0.430
**Inferior fronto-occipital fasciculus p ≤ 0.10**	-0.038 (0.034)	-1.105	0.270	1.000	0.141
**Inferior fronto-occipital fasciculus p ≤ 1.00**	-0.044 (0.035)	-1.265	0.206	1.000	0.193
**Inferior longitudinal fasciculus p ≤ 0.01**	-0.031 (0.034)	-0.916	0.360	1.000	0.094
**Inferior longitudinal fasciculus p ≤ 0.05**	-0.041 (0.034)	-1.217	0.224	1.000	0.170
**Inferior longitudinal fasciculus p ≤ 0.10**	-0.014 (0.034)	-0.416	0.677	1.000	0.020
**Inferior longitudinal fasciculus p ≤ 1.00**	-0.021 (0.035)	-0.607	0.544	1.000	0.044
**Medial lemniscus p ≤ 0.01**	0.019 (0.029)	0.643	0.520	1.000	0.036
**Medial lemniscus p ≤ 0.05**	0.016 (0.030)	0.533	0.594	1.000	0.025
**Medial lemniscus p ≤ 0.10**	0.003 (0.030)	0.116	0.907	1.000	0.001
**Medial lemniscus p ≤ 1.00**	0.038 (0.030)	1.263	0.207	1.000	0.148
**Middle cerebellar peduncle (unilateral) p ≤ 0.01**	-0.042 (0.035)	-1.217	0.224	1.000	0.178
**Middle cerebellar peduncle (unilateral) p ≤ 0.05**	-0.060 (0.035)	-1.706	0.088	1.000	0.357
**Middle cerebellar peduncle (unilateral) p ≤ 0.10**	-0.064 (0.035)	-1.832	0.067	1.000	0.414
**Middle cerebellar peduncle (unilateral) p ≤ 1.00**	-0.021 (0.036)	-0.593	0.553	1.000	0.045
**Superior longitudinal fasciculus p ≤ 0.01**	-0.010 (0.033)	-0.291	0.771	1.000	0.009
**Superior longitudinal fasciculus p ≤ 0.05**	-0.045 (0.034)	-1.321	0.187	1.000	0.199
**Superior longitudinal fasciculus p ≤ 0.10**	-0.019 (0.034)	-0.574	0.566	1.000	0.038

**Superior longitudinal fasciculus p ≤ 1.00**	-0.021 (0.035)	-0.611	0.541	1.000	0.045
**Thalamic radiation (anterior) p ≤ 0.01**	-0.061 (0.033)	-1.851	0.064	0.967	0.374
**Thalamic radiation (anterior) p ≤ 0.05**	-0.067 (0.033)	-2.005	0.045	0.679	0.447
**Thalamic radiation (anterior) p ≤ 0.10**	-0.034 (0.034)	-1.015	0.310	1.000	0.116
**Thalamic radiation (anterior) p ≤ 1.00**	-0.062 (0.034)	-1.821	0.069	1.000	0.387
**Thalamic radiation (posterior) p ≤ 0.01**	-0.020 (0.033)	-0.601	0.548	1.000	0.040
**Thalamic radiation (posterior) p ≤ 0.05**	-0.031 (0.033)	-0.926	0.355	1.000	0.095
**Thalamic radiation (posterior) p ≤ 0.10**	-0.011 (0.033)	-0.342	0.733	1.000	0.013
**Thalamic radiation (posterior) p ≤ 1.00**	-0.021 (0.034)	-0.608	0.543	1.000	0.043
**Thalamic radiation (superior) p ≤ 0.01**	0.023 (0.034)	0.671	0.502	1.000	0.051
**Thalamic radiation (superior) p ≤ 0.05**	0.008 (0.034)	0.225	0.822	1.000	0.006
**Thalamic radiation (superior) p ≤ 0.10**	0.030 (0.034)	0.882	0.378	1.000	0.090
**Thalamic radiation (superior) p ≤ 1.00**	0.063 (0.035)	1.814	0.070	1.000	0.394
**Uncinate fasciculus p ≤ 0.01**	-0.041 (0.031)	-1.318	0.188	1.000	0.172
**Uncinate fasciculus p ≤ 0.05**	-0.017 (0.032)	-0.549	0.583	1.000	0.030
**Uncinate fasciculus p ≤ 0.10**	-0.022 (0.032)	-0.694	0.488	1.000	0.049
**Uncinate fasciculus p ≤ 1.00**	-0.037 (0.032)	-1.131	0.258	1.000	0.135
**SCZ-PGRS**					
**Acoustic radiation p ≤ 0.01**	0.017 (0.031)	0.537	0.591	1.000	0.028
**Acoustic radiation p ≤ 0.05**	0.029 (0.031)	0.910	0.363	1.000	0.081
**Acoustic radiation p ≤ 0.10**	0.042 (0.031)	1.346	0.179	1.000	0.178
**Acoustic radiation p ≤ 1.00**	0.049 (0.032)	1.536	0.125	1.000	0.236
**Cingulum (cingulate gyrus) p ≤ 0.01**	-0.054 (0.031)	-1.746	0.081	1.000	0.291
**Cingulum (cingulate gyrus) p ≤ 0.05**	-0.026 (0.031)	-0.846	0.398	1.000	0.070
**Cingulum (cingulate gyrus) p ≤ 0.10**	-0.019 (0.031)	-0.618	0.537	1.000	0.037
**Cingulum (cingulate gyrus) p ≤ 1.00**	-0.012 (0.032)	-0.381	0.703	1.000	0.014
**Cingulum (parahippocampal part) p ≤ 0.01**	-0.056 (0.030)	-1.842	0.066	0.987	0.314
**Cingulum (parahippocampal part) p ≤ 0.05**	-0.075 (0.031)	-2.461	0.014	0.211	0.570
**Cingulum (parahippocampal part) p ≤ 0.10**	-0.075 (0.031)	-2.445	0.015	0.221	0.561
**Cingulum (parahippocampal part) p ≤ 1.00**	-0.055 (0.031)	-1.772	0.077	1.000	0.303
**Corticospinal tract p ≤ 0.01**	-0.001 (0.033)	-0.034	0.973	1.000	0.000
**Corticospinal tract p ≤ 0.05**	-0.004 (0.033)	-0.124	0.901	1.000	0.002
**Corticospinal tract p ≤ 0.10**	0.030 (0.033)	0.906	0.365	1.000	0.089
**Corticospinal tract p ≤ 1.00**	0.021 (0.033)	0.628	0.530	1.000	0.044
**Forceps major (unilateral) p ≤ 0.01**	0.007 (0.036)	0.192	0.848	1.000	0.005
**Forceps major (unilateral) p ≤ 0.05**	0.021 (0.036)	0.576	0.565	1.000	0.043
**Forceps major (unilateral) p ≤ 0.10**	0.044 (0.036)	1.229	0.220	1.000	0.196
**Forceps major (unilateral) p ≤ 1.00**	0.032 (0.036)	0.865	0.387	1.000	0.100
**Forceps minor (unilateral) p ≤ 0.01**	-0.044 (0.034)	-1.274	0.203	1.000	0.192
**Forceps minor (unilateral) p ≤ 0.05**	-0.014 (0.035)	-0.402	0.688	1.000	0.020
**Forceps minor (unilateral) p ≤ 0.10**	0.008 (0.035)	0.228	0.819	1.000	0.006
**Forceps minor (unilateral) p ≤ 1.00**	0.012 (0.035)	0.349	0.727	1.000	0.015
**Inferior fronto-occipital fasciculus p ≤ 0.01**	0.004 (0.034)	0.112	0.911	1.000	0.001
**Inferior fronto-occipital fasciculus p ≤ 0.05**	0.026 (0.034)	0.774	0.439	1.000	0.070
**Inferior fronto-occipital fasciculus p ≤ 0.10**	0.048 (0.034)	1.395	0.163	1.000	0.226
**Inferior fronto-occipital fasciculus p ≤ 1.00**	0.045 (0.034)	1.296	0.195	1.000	0.199
**Inferior longitudinal fasciculus p ≤ 0.01**	-0.012 (0.034)	-0.366	0.714	1.000	0.015
**Inferior longitudinal fasciculus p ≤ 0.05**	0.021 (0.034)	0.608	0.543	1.000	0.043

**Inferior longitudinal fasciculus p ≤ 0.10**	0.040 (0.034)	1.183	0.237	1.000	0.162
**Inferior longitudinal fasciculus p ≤ 1.00**	0.036 (0.034)	1.054	0.292	1.000	0.132
**Medial lemniscus p ≤ 0.01**	-0.018 (0.030)	-0.615	0.539	1.000	0.033
**Medial lemniscus p ≤ 0.05**	-0.017 (0.030)	-0.562	0.574	1.000	0.028
**Medial lemniscus p ≤ 0.10**	0.002 (0.030)	0.057	0.954	1.000	0.000
**Medial lemniscus p ≤ 1.00**	0.030 (0.030)	1.003	0.316	1.000	0.092
**Middle cerebellar peduncle (unilateral) p ≤ 0.01**	-0.001 (0.035)	-0.035	0.972	1.000	0.000
**Middle cerebellar peduncle (unilateral) p ≤ 0.05**	-0.011 (0.035)	-0.315	0.752	1.000	0.012
**Middle cerebellar peduncle (unilateral) p ≤ 0.10**	-0.017 (0.035)	-0.497	0.620	1.000	0.031
**Middle cerebellar peduncle (unilateral) p ≤ 1.00**	-0.014 (0.036)	-0.397	0.691	1.000	0.020
**Superior longitudinal fasciculus p ≤ 0.01**	-0.011 (0.034)	-0.336	0.737	1.000	0.013
**Superior longitudinal fasciculus p ≤ 0.05**	0.012 (0.034)	0.344	0.731	1.000	0.014
**Superior longitudinal fasciculus p ≤ 0.10**	0.024 (0.034)	0.717	0.474	1.000	0.059
**Superior longitudinal fasciculus p ≤ 1.00**	0.012 (0.034)	0.345	0.730	1.000	0.014
**Thalamic radiation (anterior) p ≤ 0.01**	0.024 (0.033)	0.715	0.475	1.000	0.057
**Thalamic radiation (anterior) p ≤ 0.05**	0.023 (0.034)	0.691	0.490	1.000	0.054
**Thalamic radiation (anterior) p ≤ 0.10**	0.042 (0.034)	1.243	0.214	1.000	0.174
**Thalamic radiation (anterior) p ≤ 1.00**	0.046 (0.034)	1.355	0.176	1.000	0.211
**Thalamic radiation (posterior) p ≤ 0.01**	-0.047 (0.033)	-1.411	0.159	1.000	0.219
**Thalamic radiation (posterior) p ≤ 0.05**	-0.017 (0.034)	-0.498	0.618	1.000	0.028
**Thalamic radiation (posterior) p ≤ 0.10**	0.001 (0.034)	0.038	0.970	1.000	0.000
**Thalamic radiation (posterior) p ≤ 1.00**	0.015 (0.034)	0.449	0.654	1.000	0.023
**Thalamic radiation (superior) p ≤ 0.01**	0.020 (0.034)	0.581	0.561	1.000	0.038
**Thalamic radiation (superior) p ≤ 0.05**	0.030 (0.034)	0.889	0.374	1.000	0.092
**Thalamic radiation (superior) p ≤ 0.10**	0.060 (0.034)	1.766	0.078	1.000	0.360
**Thalamic radiation (superior) p ≤ 1.00**	0.049 (0.034)	1.430	0.153	1.000	0.241
**Uncinate fasciculus p ≤ 0.01**	-0.008 (0.032)	-0.268	0.789	1.000	0.007
**Uncinate fasciculus p ≤ 0.05**	0.029 (0.032)	0.923	0.357	1.000	0.087
**Uncinate fasciculus p ≤ 0.10**	0.042 (0.032)	1.334	0.183	1.000	0.181
**Uncinate fasciculus p ≤ 1.00**	0.048 (0.032)	1.486	0.138	1.000	0.229
**BP-PGRS**					
**Acoustic radiation p ≤ 0.01**	-0.008 (0.031)	-0.260	0.795	1.000	0.007
**Acoustic radiation p ≤ 0.05**	-0.000 (0.031)	-0.011	0.991	1.000	0.000
**Acoustic radiation p ≤ 0.10**	-0.025 (0.031)	-0.793	0.428	1.000	0.061
**Acoustic radiation p ≤ 1.00**	-0.006 (0.031)	-0.207	0.836	1.000	0.004
**Cingulum (cingulate gyrus) p ≤ 0.01**	-0.038 (0.031)	-1.234	0.218	1.000	0.145
**Cingulum (cingulate gyrus) p ≤ 0.05**	-0.034 (0.031)	-1.116	0.265	1.000	0.119
**Cingulum (cingulate gyrus) p ≤ 0.10**	-0.039 (0.031)	-1.269	0.205	1.000	0.155
**Cingulum (cingulate gyrus) p ≤ 1.00**	0.000 (0.031)	0.012	0.991	1.000	0.000
**Cingulum (parahippocampal part) p ≤ 0.01**	-0.007 (0.030)	-0.237	0.813	1.000	0.005
**Cingulum (parahippocampal part) p ≤ 0.05**	-0.006 (0.030)	-0.204	0.839	1.000	0.004
**Cingulum (parahippocampal part) p ≤ 0.10**	-0.014 (0.031)	-0.447	0.655	1.000	0.019
**Cingulum (parahippocampal part) p ≤ 1.00**	0.013 (0.031)	0.428	0.669	1.000	0.017
**Corticospinal tract p ≤ 0.01**	0.008 (0.033)	0.248	0.804	1.000	0.007
**Corticospinal tract p ≤ 0.05**	0.031 (0.033)	0.941	0.347	1.000	0.094
**Corticospinal tract p ≤ 0.10**	0.017 (0.033)	0.528	0.597	1.000	0.030
**Corticospinal tract p ≤ 1.00**	0.027 (0.033)	0.823	0.411	1.000	0.073
**Forceps major (unilateral) p ≤ 0.01**	-0.003 (0.036)	-0.087	0.931	1.000	0.001

**Forceps major (unilateral) p ≤ 0.05**	0.037 (0.036)	1.028	0.304	1.000	0.135
**Forceps major (unilateral) p ≤ 0.10**	0.047 (0.036)	1.298	0.195	1.000	0.216
**Forceps major (unilateral) p ≤ 1.00**	0.035 (0.036)	0.981	0.327	1.000	0.124
**Forceps minor (unilateral) p ≤ 0.01**	-0.040 (0.034)	-1.156	0.248	1.000	0.158
**Forceps minor (unilateral) p ≤ 0.05**	-0.026 (0.034)	-0.766	0.444	1.000	0.070
**Forceps minor (unilateral) p ≤ 0.10**	-0.026 (0.035)	-0.754	0.451	1.000	0.068
**Forceps minor (unilateral) p ≤ 1.00**	-0.003 (0.035)	-0.094	0.925	1.000	0.001
**Inferior fronto-occipital fasciculus p ≤ 0.01**	-0.026 (0.034)	-0.768	0.442	1.000	0.067
**Inferior fronto-occipital fasciculus p ≤ 0.05**	-0.002 (0.034)	-0.048	0.962	1.000	0.000
**Inferior fronto-occipital fasciculus p ≤ 0.10**	-0.006 (0.034)	-0.190	0.849	1.000	0.004
**Inferior fronto-occipital fasciculus p ≤ 1.00**	0.011 (0.034)	0.330	0.741	1.000	0.013
**Inferior longitudinal fasciculus p ≤ 0.01**	-0.013 (0.034)	-0.379	0.705	1.000	0.016
**Inferior longitudinal fasciculus p ≤ 0.05**	0.010 (0.034)	0.301	0.763	1.000	0.010
**Inferior longitudinal fasciculus p ≤ 0.10**	-0.004 (0.034)	-0.132	0.895	1.000	0.002
**Inferior longitudinal fasciculus p ≤ 1.00**	-0.004 (0.034)	-0.117	0.907	1.000	0.002
**Medial lemniscus p ≤ 0.01**	0.014 (0.030)	0.459	0.647	1.000	0.018
**Medial lemniscus p ≤ 0.05**	0.050 (0.030)	1.681	0.093	1.000	0.246
**Medial lemniscus p ≤ 0.10**	0.051 (0.030)	1.717	0.086	1.000	0.259
**Medial lemniscus p ≤ 1.00**	0.040 (0.030)	1.342	0.180	1.000	0.159
**Middle cerebellar peduncle (unilateral) p ≤ 0.01**	-0.024 (0.035)	-0.691	0.490	1.000	0.058
**Middle cerebellar peduncle (unilateral) p ≤ 0.05**	0.002 (0.035)	0.051	0.960	1.000	0.000
**Middle cerebellar peduncle (unilateral) p ≤ 0.10**	-0.011 (0.035)	-0.308	0.758	1.000	0.012
**Middle cerebellar peduncle (unilateral) p ≤ 1.00**	0.003 (0.035)	0.081	0.935	1.000	0.001
**Superior longitudinal fasciculus p ≤ 0.01**	-0.037 (0.034)	-1.096	0.273	1.000	0.136
**Superior longitudinal fasciculus p ≤ 0.05**	-0.003 (0.034)	-0.095	0.924	1.000	0.001
**Superior longitudinal fasciculus p ≤ 0.10**	-0.031 (0.034)	-0.927	0.354	1.000	0.098
**Superior longitudinal fasciculus p ≤ 1.00**	-0.007 (0.034)	-0.211	0.833	1.000	0.005
**Thalamic radiation (anterior) p ≤ 0.01**	-0.013 (0.033)	-0.389	0.697	1.000	0.017
**Thalamic radiation (anterior) p ≤ 0.05**	0.002 (0.033)	0.058	0.954	1.000	0.000
**Thalamic radiation (anterior) p ≤ 0.10**	-0.004 (0.033)	-0.113	0.910	1.000	0.001
**Thalamic radiation (anterior) p ≤ 1.00**	-0.007 (0.033)	-0.216	0.829	1.000	0.005
**Thalamic radiation (posterior) p ≤ 0.01**	-0.047 (0.033)	-1.413	0.158	1.000	0.219
**Thalamic radiation (posterior) p ≤ 0.05**	-0.007 (0.033)	-0.224	0.823	1.000	0.006
**Thalamic radiation (posterior) p ≤ 0.10**	-0.007 (0.033)	-0.213	0.831	1.000	0.005
**Thalamic radiation (posterior) p ≤ 1.00**	-0.010 (0.033)	-0.305	0.761	1.000	0.010
**Thalamic radiation (superior) p ≤ 0.01**	-0.019 (0.034)	-0.570	0.569	1.000	0.037
**Thalamic radiation (superior) p ≤ 0.05**	0.018 (0.034)	0.526	0.599	1.000	0.031
**Thalamic radiation (superior) p ≤ 0.10**	0.005 (0.034)	0.157	0.875	1.000	0.003
**Thalamic radiation (superior) p ≤ 1.00**	0.007 (0.034)	0.203	0.839	1.000	0.005
**Uncinate fasciculus p ≤ 0.01**	0.001 (0.032)	0.036	0.971	1.000	0.000
**Uncinate fasciculus p ≤ 0.05**	0.020 (0.032)	0.641	0.521	1.000	0.041
**Uncinate fasciculus p ≤ 0.10**	0.012 (0.032)	0.372	0.710	1.000	0.014
**Uncinate fasciculus p ≤ 1.00**	0.033 (0.032)	1.048	0.295	1.000	0.111

FA: fractional anisotropy, WM: white matter, g: general factor, MDD: major depressive disorder, SCZ: schizophrenia, BP: bipolar disorder, PGRS: polygenic risk scores, S.D.: standard deviation. Controlled for age, age^2 and gender, genotype batch and array, and 15 MDS components. R^2 = estimate of variance explained by PGRS in %.

Table S9. Association of PGRS (MDD, SCZ and BP) at p≤ 0.5 with MD, in sample including and excluding outliers.

	Including outliers (N = 816)					Excluding outliers (N = 733)				
	Beta: z ratio (S.D.)	t statistic	p -uncorr.	p-FDR	R^2	Beta: z ratio (S.D.)	t statistic	p -uncorr	p-FDR	R^2
**MDD-PGRS**										
**Acoustic radiation**	0.052 (0.029)	1.752	0.080	0.962	0.267	0.049 (0.031)	1.602	0.110	1.000	0.242
**Cingulum (cingulate gyrus)**	0.030 (0.034)	0.895	0.371	1.000	0.090	0.017 (0.036)	0.482	0.630	1.000	0.029
**Cingulum (parahippocampal part)**	0.016 (0.031)	0.520	0.603	1.000	0.025	0.020 (0.033)	0.624	0.533	1.000	0.042
**Corticospinal tract**	0.015 (0.033)	0.451	0.652	1.000	0.022	0.004 (0.035)	0.128	0.898	1.000	0.002
**Forceps major (unilateral)**	0.003 (0.038)	0.074	0.941	1.000	0.001	-0.010 (0.040)	-0.240	0.810	1.000	0.009
**Forceps minor (unilateral)**	-0.014 (0.037)	-0.386	0.699	1.000	0.021	-0.055 (0.039)	-1.423	0.155	1.000	0.304
**Inferior fronto-occipital fasciculus**	0.071 (0.033)	2.148	0.032	0.384	0.502	0.049 (0.035)	1.416	0.157	1.000	0.240
**Inferior longitudinal fasciculus**	0.068 (0.033)	2.037	0.042	0.503	0.462	0.044 (0.035)	1.256	0.210	1.000	0.194
**Medial lemniscus**	-0.002 (0.031)	-0.051	0.959	1.000	0.000	-0.020 (0.033)	-0.620	0.536	1.000	0.041
**Middle cerebellar peduncle (unilateral)**	0.054 (0.037)	1.467	0.143	1.000	0.291	0.084 (0.038)	2.195	0.029	0.428	0.712
**Superior longitudinal fasciculus**	0.048 (0.034)	1.418	0.157	1.000	0.227	0.020 (0.036)	0.554	0.580	1.000	0.039
**Thalamic radiation (anterior)**	0.086 (0.031)	2.790	0.005	0.065 #	0.732	0.081 (0.032)	2.509	0.012	0.148	0.652
**Thalamic radiation (posterior)**	0.052 (0.032)	1.651	0.099	1.000	0.274	0.033 (0.033)	1.008	0.314	1.000	0.112
**Thalamic radiation (superior)**	0.028 (0.033)	0.850	0.396	1.000	0.077	0.012 (0.034)	0.344	0.731	1.000	0.014
**Uncinate fasciculus**	0.064 (0.032)	2.010	0.045	0.537	0.412	0.051 (0.034)	1.510	0.131	1.000	0.260
**SCZ-PGRS**										
**Acoustic radiation**	0.020 (0.030)	0.677	0.498	1.000	0.040	0.019 (0.031)	0.607	0.544	1.000	0.035
**Cingulum (cingulate gyrus)**	0.022 (0.034)	0.650	0.516	1.000	0.048	0.023 (0.036)	0.645	0.519	1.000	0.054
**Cingulum (parahippocampal part)**	0.062 (0.031)	2.006	0.045	0.542	0.378	0.046 (0.033)	1.406	0.160	1.000	0.216
**Corticospinal tract**	0.051 (0.033)	1.557	0.120	1.000	0.262	0.050 (0.035)	1.418	0.157	1.000	0.246
**Forceps major (unilateral)**	-0.045 (0.038)	-1.202	0.230	1.000	0.204	-0.042 (0.040)	-1.071	0.285	1.000	0.184
**Forceps minor (unilateral)**	-0.016 (0.037)	-0.420	0.675	1.000	0.024	-0.037 (0.039)	-0.941	0.347	1.000	0.135
**Inferior fronto-occipital fasciculus**	-0.016 (0.033)	-0.483	0.629	1.000	0.026	-0.019 (0.035)	-0.549	0.583	1.000	0.037
**Inferior longitudinal fasciculus**	-0.032 (0.034)	-0.966	0.334	1.000	0.105	-0.031 (0.035)	-0.881	0.379	1.000	0.097
**Medial lemniscus**	0.027 (0.031)	0.867	0.386	1.000	0.073	0.015 (0.033)	0.447	0.655	1.000	0.022
**Middle cerebellar peduncle (unilateral)**	0.038 (0.037)	1.031	0.303	1.000	0.143	0.029 (0.039)	0.754	0.451	1.000	0.086
**Superior longitudinal fasciculus**	-0.001 (0.034)	-0.018	0.986	1.000	0.000	-0.016 (0.036)	-0.451	0.652	1.000	0.026

**Thalamic radiation (anterior)**	0.015 (0.031)	0.470	0.639	1.000	0.021	0.018 (0.033)	0.538	0.591	1.000	0.031
**Thalamic radiation (posterior)**	0.044 (0.032)	1.387	0.166	1.000	0.196	0.029 (0.033)	0.879	0.380	1.000	0.087
**Thalamic radiation (superior)**	0.018 (0.033)	0.538	0.591	1.000	0.031	0.004 (0.035)	0.109	0.913	1.000	0.001
**Uncinate fasciculus**	-0.021 (0.032)	-0.660	0.509	1.000	0.045	-0.007 (0.034)	-0.217	0.828	1.000	0.005
**BP-PGRS**										
**Acoustic radiation**	0.011 (0.029)	0.375	0.708	1.000	0.012	-0.009 (0.031)	-0.304	0.761	1.000	0.009
**Cingulum (cingulate gyrus)**	0.018 (0.033)	0.547	0.585	1.000	0.034	0.005 (0.035)	0.139	0.889	1.000	0.002
**Cingulum (parahippocampal part)**	0.043 (0.030)	1.411	0.159	1.000	0.184	0.040 (0.033)	1.233	0.218	1.000	0.162
**Corticospinal tract**	0.029 (0.033)	0.899	0.369	1.000	0.086	0.008 (0.035)	0.229	0.819	1.000	0.006
**Forceps major (unilateral)**	-0.056 (0.037)	-1.533	0.126	1.000	0.317	-0.054 (0.039)	-1.379	0.168	1.000	0.287
**Forceps minor (unilateral)**	-0.038 (0.036)	-1.065	0.287	1.000	0.148	-0.070 (0.038)	-1.840	0.066	0.993	0.486
**Inferior fronto-occipital fasciculus**	0.011 (0.033)	0.331	0.741	1.000	0.012	-0.033 (0.035)	-0.954	0.340	1.000	0.109
**Inferior longitudinal fasciculus**	-0.005 (0.033)	-0.149	0.881	1.000	0.002	-0.037 (0.035)	-1.058	0.291	1.000	0.137
**Medial lemniscus**	-0.016 (0.031)	-0.514	0.607	1.000	0.025	-0.019 (0.033)	-0.594	0.553	1.000	0.038
**Middle cerebellar peduncle (unilateral)**	-0.047 (0.036)	-1.305	0.192	1.000	0.219	-0.036 (0.038)	-0.966	0.334	1.000	0.133
**Superior longitudinal fasciculus**	0.014 (0.033)	0.417	0.677	1.000	0.019	-0.026 (0.035)	-0.722	0.471	1.000	0.066
**Thalamic radiation (anterior)**	0.025 (0.031)	0.819	0.413	1.000	0.063	-0.013 (0.032)	-0.390	0.696	1.000	0.016
**Thalamic radiation (posterior)**	-0.010 (0.032)	-0.320	0.749	1.000	0.010	-0.037 (0.033)	-1.117	0.264	1.000	0.137
**Thalamic radiation (superior)**	0.028 (0.033)	0.856	0.392	1.000	0.077	-0.014 (0.034)	-0.418	0.676	1.000	0.021
**Uncinate fasciculus**	-0.004 (0.032)	-0.119	0.905	1.000	0.001	-0.021 (0.034)	-0.614	0.539	1.000	0.043

MDD: major depressive disorder, SCZ: schizophrenia, BP: bipolar disorder, PGRS: polygenic risk scores, uncorr.: uncorrected, FDR: false discovery rate. Controlled for age, age^2, gender, genotype batch and array,15 MDS components and intracranial volume. R^2 = estimate of variance explained by PGRS in %. # depicts trendwise associations (p-FDR < 0.10).

Table S10. Association of PGRS (MDD, SCZ or BP) at other p thresholds with MD, in sample including outliers.

	Including outliers (N= 816)				
	**Beta: z ratio (S.D.)**	**t statistic**	**p -uncorr.**	**p-FDR**	R^2
**MDD-PGRS**					
**Acoustic radiation p ≤ 0.01**	0.058 (0.029)	1.989	0.047	0.706	0.336
**Acoustic radiation p ≤ 0.05**	0.051 (0.029)	1.744	0.081	1.000	0.263
**Acoustic radiation p ≤ 0.10**	0.052 (0.030)	1.760	0.079	1.000	0.270
**Acoustic radiation p ≤ 1.00**	0.051 (0.030)	1.703	0.089	1.000	0.263
**Cingulum (cingulate gyrus) p ≤ 0.01**	0.009 (0.033)	0.284	0.776	1.000	0.009
**Cingulum (cingulate gyrus) p ≤ 0.05**	0.032 (0.034)	0.961	0.337	1.000	0.104
**Cingulum (cingulate gyrus) p ≤ 0.10**	0.037 (0.034)	1.086	0.278	1.000	0.134
**Cingulum (cingulate gyrus) p ≤ 1.00**	0.025 (0.034)	0.729	0.466	1.000	0.063
**Cingulum (parahippocampal part) p ≤ 0.01**	0.043 (0.030)	1.434	0.152	1.000	0.187
**Cingulum (parahippocampal part) p ≤ 0.05**	0.016 (0.030)	0.535	0.593	1.000	0.027
**Cingulum (parahippocampal part) p ≤ 0.10**	0.006 (0.031)	0.190	0.849	1.000	0.003
**Cingulum (parahippocampal part) p ≤ 1.00**	0.036 (0.031)	1.159	0.247	1.000	0.131
**Corticospinal tract p ≤ 0.01**	0.028 (0.032)	0.874	0.383	1.000	0.080
**Corticospinal tract p ≤ 0.05**	0.016 (0.033)	0.504	0.614	1.000	0.027
**Corticospinal tract p ≤ 0.10**	0.029 (0.033)	0.889	0.374	1.000	0.085
**Corticospinal tract p ≤ 1.00**	0.018 (0.033)	0.538	0.591	1.000	0.032
**Forceps major (unilateral) p ≤ 0.01**	0.023 (0.035)	0.644	0.520	1.000	0.052
**Forceps major (unilateral) p ≤ 0.05**	0.046 (0.036)	1.289	0.198	1.000	0.212
**Forceps major (unilateral) p ≤ 0.10**	0.040 (0.036)	1.107	0.269	1.000	0.158
**Forceps major (unilateral) p ≤ 1.00**	0.004 (0.037)	0.114	0.910	1.000	0.002
**Forceps minor (unilateral) p ≤ 0.01**	0.001 (0.035)	0.036	0.971	1.000	0.000
**Forceps minor (unilateral) p ≤ 0.05**	0.021 (0.035)	0.592	0.554	1.000	0.044
**Forceps minor (unilateral) p ≤ 0.10**	0.009 (0.035)	0.265	0.791	1.000	0.009
**Forceps minor (unilateral) p ≤ 1.00**	-0.017 (0.036)	-0.460	0.645	1.000	0.028
**Inferior fronto-occipital fasciculus p ≤ 0.01**	0.072 (0.033)	2.205	0.028	0.416	0.517
**Inferior fronto-occipital fasciculus p ≤ 0.05**	0.072 (0.033)	2.192	0.029	0.430	0.521
**Inferior fronto-occipital fasciculus p ≤ 0.10**	0.045 (0.033)	1.354	0.176	1.000	0.201
**Inferior fronto-occipital fasciculus p ≤ 1.00**	0.038 (0.034)	1.125	0.261	1.000	0.145
**Inferior longitudinal fasciculus p ≤ 0.01**	0.069 (0.033)	2.080	0.038	0.568	0.470
**Inferior longitudinal fasciculus p ≤ 0.05**	0.069 (0.033)	2.069	0.039	0.583	0.474
**Inferior longitudinal fasciculus p ≤ 0.10**	0.036 (0.033)	1.071	0.284	1.000	0.128
**Inferior longitudinal fasciculus p ≤ 1.00**	0.034 (0.034)	0.985	0.325	1.000	0.113
**Medial lemniscus p ≤ 0.01**	0.036 (0.031)	1.162	0.245	1.000	0.127
**Medial lemniscus p ≤ 0.05**	-0.003 (0.031)	-0.081	0.935	1.000	0.001
**Medial lemniscus p ≤ 0.10**	0.010 (0.031)	0.338	0.736	1.000	0.011
**Medial lemniscus p ≤ 1.00**	0.032 (0.032)	1.007	0.314	1.000	0.102
**Middle cerebellar peduncle (unilateral) p ≤ 0.01**	0.064 (0.035)	1.838	0.066	0.997	0.406
**Middle cerebellar peduncle (unilateral) p ≤ 0.05**	0.048 (0.035)	1.373	0.170	1.000	0.231
**Middle cerebellar peduncle (unilateral) p ≤ 0.10**	0.034 (0.035)	0.976	0.329	1.000	0.118
**Middle cerebellar peduncle (unilateral) p ≤ 1.00**	0.054 (0.036)	1.513	0.131	1.000	0.294
**Superior longitudinal fasciculus p ≤ 0.01**	0.029 (0.033)	0.867	0.386	1.000	0.083
**Superior longitudinal fasciculus p ≤ 0.05**	0.049 (0.034)	1.471	0.142	1.000	0.243
**Superior longitudinal fasciculus p ≤ 0.10**	0.037 (0.034)	1.103	0.270	1.000	0.138

**Superior longitudinal fasciculus p ≤ 1.00**	0.032 (0.034)	0.940	0.348	1.000	0.104
**Thalamic radiation (anterior) p ≤ 0.01**	0.084 (0.030)	2.775	0.006	0.085 #	0.710
**Thalamic radiation (anterior) p ≤ 0.05**	0.088 (0.031)	2.881	0.004	0.061 #	0.779
**Thalamic radiation (anterior) p ≤ 0.10**	0.070 (0.031)	2.280	0.023	0.343	0.493
**Thalamic radiation (anterior) p ≤ 1.00**	0.056 (0.031)	1.770	0.077	1.000	0.310
**Thalamic radiation (posterior) p ≤ 0.01**	0.056 (0.031)	1.800	0.072	1.000	0.318
**Thalamic radiation (posterior) p ≤ 0.05**	0.052 (0.032)	1.655	0.098	1.000	0.274
**Thalamic radiation (posterior) p ≤ 0.10**	0.022 (0.032)	0.688	0.491	1.000	0.048
**Thalamic radiation (posterior) p ≤ 1.00**	0.017 (0.032)	0.514	0.608	1.000	0.028
**Thalamic radiation (superior) p ≤ 0.01**	0.021 (0.032)	0.659	0.510	1.000	0.045
**Thalamic radiation (superior) p ≤ 0.05**	0.030 (0.033)	0.925	0.355	1.000	0.091
**Thalamic radiation (superior) p ≤ 0.10**	0.029 (0.033)	0.884	0.377	1.000	0.084
**Thalamic radiation (superior) p ≤ 1.00**	0.009 (0.033)	0.283	0.777	1.000	0.009
**Uncinate fasciculus p ≤ 0.01**	0.064 (0.032)	2.014	0.044	0.666	0.405
**Uncinate fasciculus p ≤ 0.05**	0.067 (0.032)	2.087	0.037	0.559	0.443
**Uncinate fasciculus p ≤ 0.10**	0.066 (0.032)	2.071	0.039	0.580	0.439
**Uncinate fasciculus p ≤ 1.00**	0.066 (0.033)	2.021	0.044	0.654	0.436
**SCZ-PGRS**					
**Acoustic radiation p ≤ 0.01**	0.029 (0.029)	0.972	0.331	1.000	0.081
**Acoustic radiation p ≤ 0.05**	0.020 (0.030)	0.675	0.500	1.000	0.040
**Acoustic radiation p ≤ 0.10**	0.017 (0.030)	0.562	0.575	1.000	0.028
**Acoustic radiation p ≤ 1.00**	0.008 (0.030)	0.263	0.792	1.000	0.006
**Cingulum (cingulate gyrus) p ≤ 0.01**	0.030 (0.033)	0.911	0.362	1.000	0.093
**Cingulum (cingulate gyrus) p ≤ 0.05**	0.019 (0.034)	0.569	0.569	1.000	0.037
**Cingulum (cingulate gyrus) p ≤ 0.10**	0.005 (0.034)	0.162	0.872	1.000	0.003
**Cingulum (cingulate gyrus) p ≤ 1.00**	-0.000 (0.034)	-0.004	0.996	1.000	0.000
**Cingulum (parahippocampal part) p ≤ 0.01**	0.015 (0.030)	0.490	0.625	1.000	0.022
**Cingulum (parahippocampal part) p ≤ 0.05**	0.061 (0.031)	1.994	0.046	0.697	0.372
**Cingulum (parahippocampal part) p ≤ 0.10**	0.067 (0.031)	2.209	0.027	0.412	0.455
**Cingulum (parahippocampal part) p ≤ 1.00**	0.062 (0.031)	1.994	0.046	0.697	0.379
**Corticospinal tract p ≤ 0.01**	0.034 (0.032)	1.058	0.291	1.000	0.118
**Corticospinal tract p ≤ 0.05**	0.049 (0.033)	1.496	0.135	1.000	0.241
**Corticospinal tract p ≤ 0.10**	0.051 (0.033)	1.569	0.117	1.000	0.264
**Corticospinal tract p ≤ 1.00**	0.054 (0.033)	1.644	0.101	1.000	0.296
**Forceps major (unilateral) p ≤ 0.01**	-0.027 (0.036)	-0.746	0.456	1.000	0.070
**Forceps major (unilateral) p ≤ 0.05**	-0.042 (0.036)	-1.175	0.240	1.000	0.178
**Forceps major (unilateral) p ≤ 0.10**	-0.063 (0.036)	-1.748	0.081	1.000	0.393
**Forceps major (unilateral) p ≤ 1.00**	-0.060 (0.036)	-1.646	0.100	1.000	0.356
**Forceps minor (unilateral) p ≤ 0.01**	0.027 (0.035)	0.766	0.444	1.000	0.073
**Forceps minor (unilateral) p ≤ 0.05**	-0.015 (0.036)	-0.431	0.667	1.000	0.024
**Forceps minor (unilateral) p ≤ 0.10**	-0.031 (0.036)	-0.877	0.381	1.000	0.097
**Forceps minor (unilateral) p ≤ 1.00**	-0.027 (0.036)	-0.740	0.459	1.000	0.071
**Inferior fronto-occipital fasciculus p ≤ 0.01**	0.009 (0.033)	0.260	0.795	1.000	0.007
**Inferior fronto-occipital fasciculus p ≤ 0.05**	-0.018 (0.033)	-0.536	0.592	1.000	0.032
**Inferior fronto-occipital fasciculus p ≤ 0.10**	-0.029 (0.033)	-0.861	0.390	1.000	0.082
**Inferior fronto-occipital fasciculus p ≤ 1.00**	-0.017 (0.034)	-0.510	0.610	1.000	0.029
**Inferior longitudinal fasciculus p ≤ 0.01**	-0.010 (0.033)	-0.316	0.752	1.000	0.011
**Inferior longitudinal fasciculus p ≤ 0.05**	-0.034 (0.034)	-1.003	0.316	1.000	0.113

**Inferior longitudinal fasciculus p ≤ 0.10**	-0.042 (0.033)	-1.255	0.210	1.000	0.177
**Inferior longitudinal fasciculus p ≤ 1.00**	-0.033 (0.034)	-0.961	0.337	1.000	0.106
**Medial lemniscus p ≤ 0.01**	0.039 (0.031)	1.278	0.201	1.000	0.155
**Medial lemniscus p ≤ 0.05**	0.028 (0.031)	0.899	0.369	1.000	0.078
**Medial lemniscus p ≤ 0.10**	0.012 (0.031)	0.386	0.699	1.000	0.014
**Medial lemniscus p ≤ 1.00**	0.014 (0.031)	0.441	0.659	1.000	0.019
**Middle cerebellar peduncle (unilateral) p ≤ 0.01**	0.030 (0.035)	0.847	0.397	1.000	0.087
**Middle cerebellar peduncle (unilateral) p ≤ 0.05**	0.034 (0.035)	0.961	0.337	1.000	0.115
**Middle cerebellar peduncle (unilateral) p ≤ 0.10**	0.040 (0.035)	1.123	0.262	1.000	0.156
**Middle cerebellar peduncle (unilateral) p ≤ 1.00**	0.040 (0.036)	1.119	0.263	1.000	0.159
**Superior longitudinal fasciculus p ≤ 0.01**	0.022 (0.033)	0.667	0.505	1.000	0.050
**Superior longitudinal fasciculus p ≤ 0.05**	-0.003 (0.034)	-0.079	0.937	1.000	0.001
**Superior longitudinal fasciculus p ≤ 0.10**	-0.006 (0.034)	-0.186	0.853	1.000	0.004
**Superior longitudinal fasciculus p ≤ 1.00**	0.008 (0.034)	0.247	0.805	1.000	0.007
**Thalamic radiation (anterior) p ≤ 0.01**	0.031 (0.031)	1.011	0.312	1.000	0.096
**Thalamic radiation (anterior) p ≤ 0.05**	0.011 (0.031)	0.365	0.715	1.000	0.013
**Thalamic radiation (anterior) p ≤ 0.10**	0.008 (0.031)	0.273	0.785	1.000	0.007
**Thalamic radiation (anterior) p ≤ 1.00**	0.004 (0.031)	0.141	0.888	1.000	0.002
**Thalamic radiation (posterior) p ≤ 0.01**	0.069 (0.031)	2.182	0.029	0.440	0.472
**Thalamic radiation (posterior) p ≤ 0.05**	0.044 (0.032)	1.377	0.169	1.000	0.192
**Thalamic radiation (posterior) p ≤ 0.10**	0.033 (0.032)	1.028	0.304	1.000	0.107
**Thalamic radiation (posterior) p ≤ 1.00**	0.023 (0.032)	0.722	0.471	1.000	0.054
**Thalamic radiation (superior) p ≤ 0.01**	0.037 (0.032)	1.154	0.249	1.000	0.140
**Thalamic radiation (superior) p ≤ 0.05**	0.015 (0.033)	0.450	0.653	1.000	0.022
**Thalamic radiation (superior) p ≤ 0.10**	0.011 (0.033)	0.327	0.744	1.000	0.011
**Thalamic radiation (superior) p ≤ 1.00**	0.005 (0.033)	0.163	0.870	1.000	0.003
**Uncinate fasciculus p ≤ 0.01**	-0.005 (0.032)	-0.164	0.870	1.000	0.003
**Uncinate fasciculus p ≤ 0.05**	-0.024 (0.032)	-0.741	0.459	1.000	0.057
**Uncinate fasciculus p ≤ 0.10**	-0.024 (0.032)	-0.746	0.456	1.000	0.057
**Uncinate fasciculus p ≤ 1.00**	-0.018 (0.032)	-0.565	0.572	1.000	0.034
**BP-PGRS**					
**Acoustic radiation p ≤ 0.01**	0.034 (0.029)	1.173	0.241	1.000	0.118
**Acoustic radiation p ≤ 0.05**	0.009 (0.029)	0.316	0.752	1.000	0.009
**Acoustic radiation p ≤ 0.10**	0.011 (0.029)	0.358	0.721	1.000	0.011
**Acoustic radiation p ≤ 1.00**	0.004 (0.030)	0.124	0.901	1.000	0.001
**Cingulum (cingulate gyrus) p ≤ 0.01**	0.003 (0.033)	0.092	0.926	1.000	0.001
**Cingulum (cingulate gyrus) p ≤ 0.05**	0.015 (0.033)	0.453	0.651	1.000	0.023
**Cingulum (cingulate gyrus) p ≤ 0.10**	0.021 (0.034)	0.628	0.530	1.000	0.044
**Cingulum (cingulate gyrus) p ≤ 1.00**	-0.005 (0.034)	-0.152	0.879	1.000	0.003
**Cingulum (parahippocampal part) p ≤ 0.01**	0.031 (0.030)	1.008	0.314	1.000	0.093
**Cingulum (parahippocampal part) p ≤ 0.05**	0.044 (0.030)	1.437	0.151	1.000	0.189
**Cingulum (parahippocampal part) p ≤ 0.10**	0.022 (0.030)	0.717	0.474	1.000	0.048
**Cingulum (parahippocampal part) p ≤ 1.00**	0.001 (0.031)	0.038	0.970	1.000	0.000
**Corticospinal tract p ≤ 0.01**	0.064 (0.032)	1.964	0.050	0.749	0.405
**Corticospinal tract p ≤ 0.05**	0.028 (0.032)	0.857	0.392	1.000	0.077
**Corticospinal tract p ≤ 0.10**	0.047 (0.033)	1.457	0.145	1.000	0.225
**Corticospinal tract p ≤ 1.00**	0.036 (0.033)	1.111	0.267	1.000	0.132
**Forceps major (unilateral) p ≤ 0.01**	-0.005 (0.036)	-0.151	0.880	1.000	0.003

**Forceps major (unilateral) p ≤ 0.05**	-0.043 (0.036)	-1.218	0.224	1.000	0.187
**Forceps major (unilateral) p ≤ 0.10**	-0.080 (0.036)	-2.234	0.026	0.387	0.632
**Forceps major (unilateral) p ≤ 1.00**	-0.063 (0.036)	-1.757	0.079	1.000	0.394
**Forceps minor (unilateral) p ≤ 0.01**	0.022 (0.035)	0.636	0.525	1.000	0.050
**Forceps minor (unilateral) p ≤ 0.05**	0.006 (0.035)	0.156	0.876	1.000	0.003
**Forceps minor (unilateral) p ≤ 0.10**	-0.010 (0.035)	-0.282	0.778	1.000	0.010
**Forceps minor (unilateral) p ≤ 1.00**	-0.045 (0.035)	-1.258	0.209	1.000	0.198
**Inferior fronto-occipital fasciculus p ≤ 0.01**	0.031 (0.033)	0.934	0.350	1.000	0.094
**Inferior fronto-occipital fasciculus p ≤ 0.05**	0.009 (0.033)	0.266	0.790	1.000	0.008
**Inferior fronto-occipital fasciculus p ≤ 0.10**	-0.001 (0.033)	-0.041	0.967	1.000	0.000
**Inferior fronto-occipital fasciculus p ≤ 1.00**	-0.016 (0.033)	-0.473	0.636	1.000	0.024
**Inferior longitudinal fasciculus p ≤ 0.01**	0.020 (0.033)	0.602	0.547	1.000	0.040
**Inferior longitudinal fasciculus p ≤ 0.05**	-0.006 (0.033)	-0.194	0.847	1.000	0.004
**Inferior longitudinal fasciculus p ≤ 0.10**	-0.014 (0.033)	-0.423	0.673	1.000	0.020
**Inferior longitudinal fasciculus p ≤ 1.00**	-0.018 (0.033)	-0.551	0.582	1.000	0.034
**Medial lemniscus p ≤ 0.01**	0.019 (0.031)	0.633	0.527	1.000	0.038
**Medial lemniscus p ≤ 0.05**	-0.015 (0.031)	-0.491	0.623	1.000	0.023
**Medial lemniscus p ≤ 0.10**	-0.010 (0.031)	-0.324	0.746	1.000	0.010
**Medial lemniscus p ≤ 1.00**	-0.000 (0.031)	-0.003	0.998	1.000	0.000
**Middle cerebellar peduncle (unilateral) p ≤ 0.01**	-0.006 (0.035)	-0.160	0.873	1.000	0.003
**Middle cerebellar peduncle (unilateral) p ≤ 0.05**	-0.025 (0.035)	-0.722	0.470	1.000	0.063
**Middle cerebellar peduncle (unilateral) p ≤ 0.10**	-0.019 (0.035)	-0.534	0.593	1.000	0.035
**Middle cerebellar peduncle (unilateral) p ≤ 1.00**	-0.026 (0.035)	-0.731	0.465	1.000	0.066
**Superior longitudinal fasciculus p ≤ 0.01**	0.028 (0.033)	0.846	0.398	1.000	0.080
**Superior longitudinal fasciculus p ≤ 0.05**	0.012 (0.033)	0.358	0.721	1.000	0.014
**Superior longitudinal fasciculus p ≤ 0.10**	0.021 (0.033)	0.617	0.537	1.000	0.043
**Superior longitudinal fasciculus p ≤ 1.00**	-0.006 (0.034)	-0.185	0.853	1.000	0.004
**Thalamic radiation (anterior) p ≤ 0.01**	0.018 (0.031)	0.599	0.549	1.000	0.034
**Thalamic radiation (anterior) p ≤ 0.05**	0.023 (0.031)	0.753	0.452	1.000	0.053
**Thalamic radiation (anterior) p ≤ 0.10**	0.015 (0.031)	0.489	0.625	1.000	0.023
**Thalamic radiation (anterior) p ≤ 1.00**	0.014 (0.031)	0.459	0.647	1.000	0.020
**Thalamic radiation (posterior) p ≤ 0.01**	0.033 (0.032)	1.036	0.300	1.000	0.107
**Thalamic radiation (posterior) p ≤ 0.05**	-0.011 (0.032)	-0.353	0.724	1.000	0.012
**Thalamic radiation (posterior) p ≤ 0.10**	-0.025 (0.032)	-0.799	0.425	1.000	0.064
**Thalamic radiation (posterior) p ≤ 1.00**	-0.025 (0.032)	-0.793	0.428	1.000	0.063
**Thalamic radiation (superior) p ≤ 0.01**	0.032 (0.032)	0.994	0.321	1.000	0.104
**Thalamic radiation (superior) p ≤ 0.05**	0.025 (0.032)	0.771	0.441	1.000	0.063
**Thalamic radiation (superior) p ≤ 0.10**	0.022 (0.033)	0.671	0.502	1.000	0.048
**Thalamic radiation (superior) p ≤ 1.00**	0.007 (0.033)	0.225	0.822	1.000	0.005
**Uncinate fasciculus p ≤ 0.01**	-0.012 (0.032)	-0.368	0.713	1.000	0.014
**Uncinate fasciculus p ≤ 0.05**	-0.004 (0.032)	-0.141	0.888	1.000	0.002
**Uncinate fasciculus p ≤ 0.10**	-0.009 (0.032)	-0.297	0.767	1.000	0.009
**Uncinate fasciculus p ≤ 1.00**	-0.016 (0.032)	-0.492	0.623	1.000	0.025

MD: mean diffusivity, WM: white matter, g: general factor, MDD: major depressive disorder, SCZ: schizophrenia, BP: bipolar disorder, PGRS: polygenic risk scores. Controlled for age, age^2, gender, genotype batch and array, and 15 MDS components. R^2 = estimate of variance explained by PGRS in %. # depicts trendwise associations (p-FDR < 0.10).

Results - Age-related effects in structural brain measures

Table S11. Association between age and total grey matter, white matter and cerebrospinal fluid. in sample including and excluding outliers.

	**Including outliers (N = 978)**				**Excluding outliers (N = 892)**			
	**Beta: z ratio (S.D.)**	**t statistic**	**p -uncorr.**	**p-FDR**	**Beta: z ratio (S.D.)**	**t statistic**	**p -uncorr**	**p-FDR**
**Age**								
**Grey matter volume**	-0.402 (0.026)	-15.537	8.366×10^{-49}	2.51×10^{-48} ***	-0.394 (0.027)	-14.329	4.730×10^{-42}	1.419×10^{-41} ***
**White matter volume**	-0.187 (0.026)	-7.282	6.773×10^{-13}	2.032×10^{-12} ***	-0.181 (0.027)	-6.698	3.752×10^{-11}	1.125×10^{-10} ***
**CSF volume**	0.356 (0.028)	12.651	4.472×10^{-34}	1.342×10^{-33} ***	0.371 (0.029)	12.766	2.157×10^{-34}	6.472×10^{-34} ***

CSF: cerebrospinal fluid, uncorr.: uncorrected. FDR: false discovery rate. Controlled for gender. *** depicts significant associations (p-FDR < 0.001).

Table S12. Association between age and subcortical volumes, in sample including and excluding outliers.

	**Including outliers (N = 978)**				**Excluding outliers (N = 892)**			
	**Beta: z ratio (S.D.)**	**t statistic**	**p -uncorr.**	**p-FDR**	**Beta: z ratio (S.D.)**	**t statistic**	**p -uncorr**	**p-FDR**
**Age**								
**Caudate**	-0.185 (0.029)	-6.362	3.056×10^{-10}	2.139×10^{-09} ***	-0.166 (0.031)	-5.375	9.803×10^{-8}	6.862×10^{-7} ***
**Hippocampus**	-0.278 (0.026)	-10.549	1.042×10^{-24}	7.295×10^{-24} ***	-0.260 (0.028)	-9.352	6.791×10^{-20}	4.754×10^{-19} ***
**Pallidum**	-0.192 (0.028)	-6.959	6.297×10^{-12}	4.408×10^{-11} ***	-0.158 (0.029)	-5.479	5.556×10^{-8}	3.889×10^{-7} ***
**Thalamus**	-0.392 (0.026)	-14.948	1.216×10^{-45}	8.511×10^{-45} ***	-0.376 (0.028)	-13.520	5.141×10^{-38}	3.599×10^{-37} ***
**Amygdala**	-0.042 (0.026)	-1.639	0.102	0.711	-0.015 (0.027)	-0.548	0.58	1.00
**Nucleus accumbens**	-0.321 (0.027)	-12.023	3.795×10^{-31}	2.656×10^{-30} ***	-0.279 (0.028)	-9.867	7.312×10^{-22}	5.118×10^{-21} ***
**Putamen**	-0.376 (0.025)	-15.132	1.274×10^{-46}	8.919×10^{-46} ***	-0.360 (0.026)	-13.642	1.290×10^{-38}	9.030×10^{-38} ***

Uncorr.: uncorrected, FDR: false discovery rate. Controlled for gender, and side of hemisphere. *** depicts significant associations (p-FDR < 0.001).

Table S13. Association between age and FA, in sample including and excluding outliers.

	Including outliers (N = 816)				Excluding outliers (N = 733)			
	**Beta: z ratio (S.D.)**	**t statistic**	**p -uncorr.**	**p-FDR**	**Beta: z ratio (S.D.)**	**t statistic**	**p -uncorr**	**p-FDR**
**Age**								
**Acoustic radiation**	-0.080 (0.031)	-2.604	0.009	0.141	-0.050 (0.032)	-1.558	0.120	1.000
**Cingulum (cingulate gyrus)**	-0.151 (0.030)	-4.997	7.132×10^{-7}	1.070×10^{-5} ***	-0.118 (0.032)	-3.715	2.189×10^{-4}	3.284×10^{-3} **
**Cingulum (parahippocampal part)**	-0.025 (0.030)	-0.838	0.402	1.000	-0.026 (0.032)	-0.800	0.424	1.000
**Corticospinal tract**	-0.042 (0.032)	-1.321	0.187	1.000	-0.033 (0.034)	-0.994	0.321	1.000
**Forceps major (unilateral)**	-0.039 (0.035)	-1.104	0.270	1.000	-0.016 (0.037)	-0.419	0.675	1.000
**Forceps minor (unilateral)**	-0.249 (0.034)	-7.325	5.762×10^{-13}	8.642×10^{-12} ***	-0.214 (0.036)	-5.933	4.594×10^{-09}	6.891×10^{-8} ***
**Inferior fronto-occipital fasciculus**	-0.189 (0.033)	-5.687	1.806×10^{-8}	2.709×10^{-7} ***	-0.173 (0.035)	-4.950	9.207×10^{-7}	1.381×10^{-5} ***
**Inferior longitudinal fasciculus**	-0.227 (0.033)	-6.865	1.316×10^{-11}	1.974×10^{-10} ***	-0.218 (0.035)	-6.268	6.254 x 10-10	9.382×10^{-9} ***
**Medial lemniscus**	0.016 (0.029)	0.564	0.573	1.000	0.015 (0.031)	0.500	0.618	1.000
**Middle cerebellar peduncle (unilateral)**	-0.030 (0.034)	-0.890	0.374	1.000	-0.002 (0.036)	-0.069	0.945	1.000
**Superior longitudinal fasciculus**	-0.224 (0.033)	-6.774	2.400×10^{-11}	3.600×10^{-10} ***	-0.182 (0.035)	-5.220	2.329×10^{-7}	3.494×10^{-6} ***
**Thalamic radiation (anterior)**	-0.226 (0.033)	-6.898	1.060×10^{-11}	1.590×10^{-10} ***	-0.196 (0.035)	-5.654	2.252×10^{-8}	3.379×10^{-7} ***
**Thalamic radiation (posterior)**	-0.144 (0.033)	-4.417	1.137×10^{-5}	1.706×10^{-4} ***	-0.140 (0.034)	-4.108	4.436×10^{-5}	6.654×10^{-4} ***
**Thalamic radiation (superior)**	-0.024 (0.033)	-0.731	0.465	1.000	0.006 (0.035)	0.167	0.867	1.000
**Uncinate fasciculus**	-0.201 (0.031)	-6.492	1.469×10^{-10}	2.203×10^{-9} ***	-0.186 (0.032)	-5.747	1.335×10^{-8}	2.003×10^{-7} ***

FA: fractional anisotropy, uncorr.: uncorrected, FDR: false discovery rate. Controlled for gender, and side of hemisphere. Asterisks depict significant associations (***: p-FDR < 0.001, **: p-FDR < 0.01).

Table S14. Association between age and MD, in sample including and excluding outliers.

	Including outliers (N= 816)				Excluding outliers (N= 733)			
	Beta: z ratio (S.D.)	t statistic	p -uncorr.	p-FDR	Beta: z ratio (S.D.)	t statistic	p -uncorr	p-FDR
**Acoustic radiation**	0.042 (0.029)	1.451	0.147	1.000	0.015 (0.030)	0.511	0.609	1.000
**Cingulum (cingulate gyrus)**	0.122 (0.033)	3.705	2.253×10^{-4}	0.003 **	0.086 (0.035)	2.453	0.014	0.216
**Cingulum (parahippocampal part)**	0.163 (0.030)	5.404	8.574×10^{-8}	1.286×10^{-6} ***	0.186 (0.032)	5.761	1.230 x 10-8	1.845×10^{-7} ***
**Corticospinal tract**	0.165 (0.032)	5.151	3.255×10^{-7}	4.882×10^{-6} ***	0.134 (0.034)	3.939	8.969 x 10-5	0.001 **
**Forceps major (unilateral)**	0.081 (0.035)	2.314	0.021	0.313	0.064 (0.037)	1.741	0.082	1.000
**Forceps minor (unilateral)**	0.165 (0.035)	4.741	2.506 x 10-6	3.759×10^{-5} ***	0.132 (0.037)	3.574	3.752 x 10-4	0.006 **
**Inferior fronto-occipital fasciculus**	0.311 (0.032)	9.646	6.423 x 10-21	9.635×10^{-20} ***	0.304 (0.034)	8.979	2.298 x 10-18	3.448×10^{-17} ***
**Inferior longitudinal fasciculus**	0.279 (0.033)	8.562	5.486 x 10-17	8.229×10^{-16} ***	0.273 (0.034)	7.995	5.084 x 10-15	7.627×10^{-14} ***
**Medial lemniscus**	0.041 (0.030)	1.345	0.179	1.000	0.042 (0.032)	1.313	0.190	1.000
**Middle cerebellar peduncle (unilateral)**	0.160 (0.035)	4.630	4.248 x 10-6	6.373×10^{-5} ***	0.167 (0.036)	4.586	5.315 x 10-6	7.972×10^{-5} ***
**Superior longitudinal fasciculus**	0.278 (0.033)	8.424	1.644 x 10-16	2.466×10^{-15} ***	0.244 (0.035)	6.988	6.295 x 10-12	9.442×10^{-11} ***
**Thalamic radiation (anterior)**	0.458 (0.030)	15.168	6.058 x 10-46	9.087×10^{-45} ***	0.462 (0.032)	14.557	2.462 x 10-42	3.693×10^{-41} ***
**Thalamic radiation (posterior)**	0.308 (0.031)	9.897	6.956 x 10-22	1.043×10^{-20} ***	0.320 (0.033)	9.828	1.711 x 10-21	2.567×10^{-20} ***
**Thalamic radiation (superior)**	0.333 (0.032)	10.367	9.806 x 10-24	1.471×10^{-22} ***	0.322 (0.034)	9.503	2.847 x 10-20	4.270×10^{-19} ***
**Uncinate fasciculus**	0.336 (0.031)	10.759	2.480 x 10-25	3.720×10^{-24} ***	0.323 (0.033)	9.794	2.313 x 10-21	3.469×10^{-20} ***

MD: mean diffusivity, uncorr.: uncorrected, FDR: false discovery rate. Controlled for gender, and side of hemisphere. Asterisks depict significant associations (***: p-FDR < 0.001, **: p-FDR < 0.01).

Table S15. Mean and standard deviation values for subcortical volumes, in sample including and excluding outliers.

	Including outliers (N = 978)		Excluding outliers (N = 892)	
	**Mean**	**S.D.**	**Mean**	**S.D.**
**GM volume**	620,527.494	55,455.483	618,025.136	53,392.164
**WM volume**	554,146.653	60,866.465	550,290.295	58,948.939
**CSF volume**	3,4750.004	16,244.125	33145.856	13,181.973
**Caudate left**	3,410.982	429.658	3,389.788	406.085
**Caudate right**	3,579.677	446.418	3,556.467	412.049
**Hippocampus left**	3,800.842	475.621	3,805.887	437.867
**Hippocampus right**	3,948.889	488.068	3,947.941	457.418
**Pallidum left**	1,752.425	243.127	1,744.527	224.818
**Pallidum right**	1,806.454	249.077	1,793.626	218.861
**Thalamus left**	7,820.510	755.085	7,800.398	721.807
**Thalamus right**	7,632.403	737.252	7,606.157	704.516
**Amygdala left**	1,268.328	254.551	1,263.684	243.473
**Amygdala right**	1,248.264	271.467	1,241.337	258.325
**Nucleus accumbens left**	511.629	119.327	5,11.833	112.243
**Nucleus accumbens right**	404.828	113.003	406.142	107.994
**Putamen left**	4,819.355	596.686	4,807.377	561.281
**Putamen right**	4,866.548	574.638	4,847.305	545.478

GM: grey matter, WM: white matter, S.D.: standard deviation

Table S16. Mean and standard deviation FA and MD values, in sample including and excluding outliers.

	Including outliers (N = 816)				Excluding outliers (N = 733)			
	FA		MD		FA		MD	
	Mean	S.D.	Mean	S.D.	Mean	S.D.	Mean	S.D.
**Acoustic radiation left**	0.425	0.023	7.797E-04	3.853E-05	0.426	0.022	7.787E-04	3.792E-05
**Acoustic radiation right**	0.416	0.022	7.822E-04	3.682E-05	0.416	0.021	7.801E-04	3.485E-04
**Thalamic radiation (anterior) left**	0.402	0.019	7.728E-04	3.301E-05	0.404	0.017	7.697E-04	2.940E-04
**Thalamic radiation (anterior) right**	0.395	0.018	7.730E-04	3.444E-05	0.396	0.017	7.696E-04	3.017E-05
**Cingulum (cingulate gyrus) left**	0.538	0.034	7.532E-04	2.806E-06	0.539	0.033	7.525E-04	2.670E-04
**Cingulum (cingulate gyrus) right**	0.498	0.033	7.571E-04	2.679E-05	0.499	0.032	7.566E-04	2.607E-04
**Corticospinal tract left**	0.548	0.021	7.700E-04	2.373E-05	0.549	0.020	7.685E-04	2.276E-04
**Corticospinal tract right**	0.542	0.023	7.745E-04	2.380E-05	0.543	0.022	7.731E-04	2.284E-04
**Forceps major (unilateral) left**	0.586	0.027	8.917E-04	5.085E-05	0.588	0.025	8.891E-04	4.955E-04
**Forceps major (unilateral) right**	0.468	0.021	8.293E-04	3.404E-05	0.469	0.019	8.274E-04	3.161E-04
**Inferior fronto-occipital fasciculus left**	0.479	0.022	8.007E-04	3.062E-05	0.481	0.020	7.979E-04	2.739E-04
**Inferior fronto-occipital fasciculus right**	0.467	0.021	8.087E-04	3.076E-05	0.469	0.019	8.062E-04	2.770E-04
**Inferior longitudinal fasciculus left**	0.463	0.021	8.107E-04	3.239E-05	0.465	0.019	8.082E-04	2.997E-04
**Inferior longitudinal fasciculus right**	0.453	0.019	8.194E-04	3.174E-05	0.455	0.018	8.164E-04	2.850E-04
**Medial lemniscus left**	0.417	0.023	9.055E-04	3.852E-05	0.418	0.023	9.048E-04	3.792E-04
**Medial lemniscus right**	0.420	0.023	9.080E-04	3.820E-05	0.421	0.023	9.079E-04	3.761E-04
**Middle cerebellar peduncle (unilateral)**	0.475	0.034	7.748E-04	7.731E-05	0.477	0.032	7.706E-04	6.510E-04
**Cingulum (parahippocampal part) left**	0.311	0.032	8.785E-04	6.482E-05	0.312	0.031	8.754E-04	6.185E-04
**Cingulum (parahippocampal part) right**	0.309	0.034	8.893E-04	6.440E-05	0.309	0.034	8.874E-04	6.364E-04
**Thalamic radiation (posterior) left**	0.458	0.020	8.399E-04	3.782E-05	0.460	0.018	8.366E-04	3.411E-04
**Thalamic radiation (posterior) right**	0.454	0.021	8.446E-04	3.948E-05	0.456	0.019	8.409E-04	3.468E-04
**Superior longitudinal fasciculus left**	0.444	0.021	7.402E-04	3.028E-05	0.445	0.019	7.374E-04	2.712E-04
**Superior longitudinal fasciculus right**	0.427	0.020	7.509E-04	3.016E-05	0.428	0.018	7.483E-04	2.680E-04
**Thalamic radiation (superior) left**	0.423	0.018	7.550E-04	2.628E-05	0.424	0.017	7.527E-04	2.381E-04
**Thalamic radiation (superior) right**	0.422	0.018	7.517E-04	2.849E-05	0.423	0.017	7.495E-04	2.601E-05
**Uncinate fasciculus left**	0.394	0.025	7.893E-04	3.988E-05	0.395	0.024	7.870E-04	3.769E-04
**Uncinate fasciculus right**	0.393	0.022	7.926E-04	3.206E-05	0.394	0.021	7.905E-04	3.010E-04

WM: white matter, MD: mean diffusivity, FA: fractional anisotropy, S.D.: standard deviation

References

1. Miller, K. L. *et al.* Multimodal population brain imaging in the UK Biobank prospective epidemiological study. *Nat. Neurosci.* **19,** (2016).
2. Jenkinson, M., Beckmann, C. F., Behrens, T. E. J., Woolrich, M. W. & Smith, S. M. Fsl. *Neuroimage* **62,** 782–790 (2012).
3. Smith, S. M. Fast robust automated brain extraction. *Hum. Brain Mapp.* **17,** 143–155 (2002).
4. Jenkinson, M. & Smith, S. A global optimisation method for robust affine registration of brain images. *Med. Image Anal.* **5,** 143–156 (2001).
5. Jenkinson, M., Bannister, P., Brady, M. & Smith, S. Improved optimization for the robust and accurate linear registration and motion correction of brain images. *Neuroimage* **17,** 825–841 (2002).
6. Andersson, J. L. R., Jenkinson, M. & Smith, S. M. Non-linear optimisation. FMRIB technical report TR07JA1. *In Pract.* 16 (2007).
7. Andersson, J. L. R., Jenkinson, M. & Smith, S. Non-linear registration aka Spatial normalisation FMRIB Technial Report TR07JA2. *In Pract.* 22 (2007).
8. Patenaude, B., Smith, S. M., Kennedy, D. & Jenkinson, M. NIH Public Access. *Neuroimage* **56,** 907–922 (2012).
9. Behrens, T. E. J., Berg, H. J., Jbabdi, S., Rushworth, M. F. S. & Woolrich, M. W. Probabilistic diffusion tractography with multiple fibre orientations: What can we gain? *Neuroimage* **34,** 144–155 (2007).
10. Groot, M. De. *Cross-Subject Image Analysis in Diffusion Brain MRI Marius de Groot*.
11. Basser, P. J., Mattiello, J. & LeBihan, D. MR diffusion tensor spectroscopy and imaging. *Biophys. J.* **66,** 259–67 (1994).
12. Pierpaoli, Carlo, et al. Diffusion tensor MR imaging of the human brain. 259–267 (1996).
13. Cercignani, M., Inglese, M., Pagani, E., Comi, G. & Filippi, M. Mean Diffusivity and Fractional Anisotropy Histograms of Patients with Multiple Sclerosis. *AJNR Am J Neuroradiol.* **22,** 952–958 (2001).
14. Jones, D. K., Knösche, T. R. & Turner, R. White matter integrity, fiber count, and other fallacies: the do’s and don’ts of diffusion MRI. *Neuroimage* **73,** 239–54 (2013).
